# Simultaneous Tunneling Control in Conformer-Specific Reactions

**DOI:** 10.1021/jacs.2c09026

**Published:** 2022-11-02

**Authors:** Cláudio M. Nunes, José P. L. Roque, Srinivas Doddipatla, Samuel A. Wood, Robert J. McMahon, Rui Fausto

**Affiliations:** † University of Coimbra, CQC-IMS, Department of Chemistry, 3004-535 Coimbra, Portugal; ‡ Department of Chemistry, University of Wisconsin−Madison, Madison, Wisconsin 53706-1322, United States

## Abstract

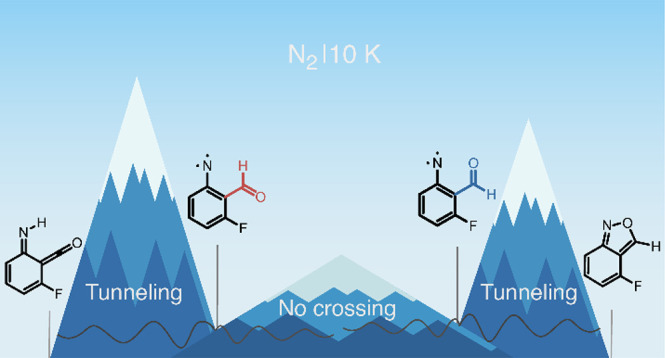

We present here a new example of chemical reactivity governed by quantum tunneling, which also highlights the limitations of the classical theories. The *syn* and *anti* conformers of a triplet 2-formylphenylnitrene, generated in a nitrogen matrix, were found to spontaneously rearrange to the corresponding 2,1-benzisoxazole and imino-ketene, respectively. The kinetics of both transformations were measured at 10 and 20 K and found to be temperature-independent, providing clear evidence of concomitant tunneling reactions (heavy-atom and H-atom). Computations confirm the existence of these tunneling reaction pathways. Although the energy barrier between the nitrene conformers is lower than any of the observed reactions, no conformational interconversion was observed. These results demonstrate an unprecedented case of simultaneous tunneling control in conformer-specific reactions of the same chemical species. The product outcome is impossible to be rationalized by the conventional kinetic or thermodynamic control.

## Introduction

Tunneling is a quantum mechanical phenomenon that describes how particles permeate through potential energy barriers.^1,2^ The occurrence of tunneling in chemical reactions used to be largely ignored, and the conceptual framework to understand chemical reactivity has been inferred from the transition state theory (TST), which assumes that nuclei behave according to classical mechanics.^3,4^ For many years, tunneling relevance to chemical reactivity has only been acknowledged in the field of chemical kinetics, where tunneling appears as a correction factor in the TST calculations of rate constants (in particular for reactions involving the transfer of hydrogen atoms or ions).^5,6^ However, recent evidence has shown that tunneling not only is more common than previously thought (even occurring for reactions involving the motion of heavy atoms such as carbon) but also can have profound consequences on the chemical reaction outcome, casting doubts on the validity of the TST principles.^7−21^

**Scheme 1:**
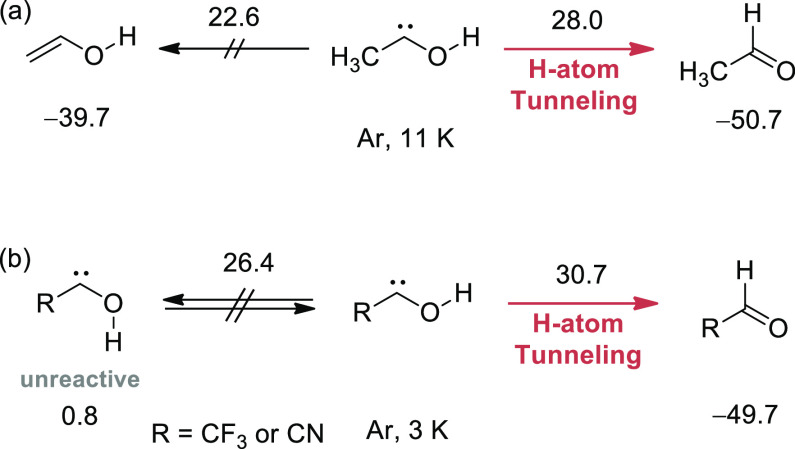
Tunneling Control (Panel a) and Conformer-Specific Tunneling (Panel b) Reactions Observed for Hydroxycarbene Derivatives by Matrix Isolation Spectroscopy The numbers indicate the relative energies Δ*H*_0K_ (in kcal mol^–1^) computed according to the approach reported in ref (22) (panel a) and ref (36) (panel b; only for R = CF_3_).

In this context, Schreiner et al. revealed in 2011 that the tunneling reaction of methylhydroxycarbene in cryogenic matrices leads exclusively to acetaldehyde, whose reaction path faces a higher barrier than the alternative route to vinyl alcohol and coined a term for this new reactivity paradigm as tunneling control (Scheme 1a).^11,22^ Because classical thermal over-the-barrier processes are hindered at cryogenic temperatures (e.g., 3–15 K), chemical transformations discovered under such conditions, with rates insensible to the increase of temperature, constitute distinctive examples of tunneling from the vibrational ground state.^17^ Apart from the observed [1,2]H-shift reactions of hydroxycarbenes to the corresponding aldehydes,^11,23^ other similar examples of tunneling control were identified^24,25^ for the thiol-thione tautomerization of thiourea,^26,27^ for the C–H insertion of *tert*-butylchlorocarbene to dimethylchlorocyclopropane^11,28^ and for the rearrangement of ketene to isoxazolone isomeric forms of *o*-nitrobenzaldehyde.^29^ Interestingly, the last two examples demonstrated that tunneling control even dictates the formation of products impossible to rationalize by either kinetic or thermodynamic control, concepts that arise from TST to explain the chemical reactivity and selectivity. In addition, a few examples of reactions directed by tunneling control at low temperatures have also been predicted computationally,^30,31^ including a singular case dominated by heavy-atom motions—the planar bond shifting in [16]annulene.^32^ Outside of the cryogenic temperature ranges, the first evidence of tunneling control directing new pathways in catalytic reactions also has been recently reported,^33−35^ a testimony that the above-mentioned breakthroughs bring new conceptual frameworks that are promising for the discovery of new chemical transformations and improved reaction planning.

Investigations exploring pure tunneling reactions under cryogenic conditions also began to reveal how tunneling reactivity is dependent on the conformations of the reactant molecules.^37,38^ Groundbreaking examples of conformer-specific H-atom tunneling reactions were reported for the trifluoromethyl- and cyanohydroxycarbenes, where both the *syn* and *anti* OH conformers were isolated for the first time. It was observed that the *syn* undergoes [1,2]H-shift tunneling, while the *anti* remains unreactive (Scheme 1b).^36,39^ These results constitute particular examples where the Curtin–Hammett principle is not followed or applicable. In a pioneering contribution to the field, we have recently demonstrated how to switch-on a tunneling reaction by conformational control using external radiation. The OH moiety in the vicinity of a nitrene center was manipulated from *anti* to *syn* orientation, by selective vibrational excitation at the 2ν(OH) frequency (near-IR light), triggering in this way the corresponding H-atom tunneling reaction.^40^ Moreover, we have shown that the conformation of an aldehyde moiety (*syn* or *anti*) in the vicinity of a nitrene center can give access to different tunneling reactions (although such a discovery regards different nitrene derivatives).^41,42^

**Scheme 2:**
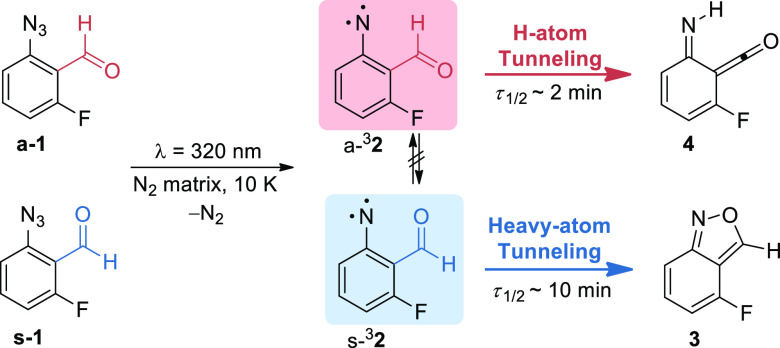
Summary of the Simultaneous Tunneling Control Reactions Observed in the Different Conformers of Triplet 2-Formyl-3-Fluorophenylnitrene (**a-**^**3**^**2** and **s-**^**3**^**2**) Generated in Nitrogen Matrices (10 or 20 K)

Pursuing advances in the understanding of tunneling reactivity and its dependence on the molecular conformations, we demonstrate here an unprecedented case where both conformers of the same species undergo, simultaneously, tunneling reactions to distinct rearrangement products.^43^ Namely, the conformers of 2-formyl-3-fluorophenylnitrene **s-**^**3**^**2** and **a-**^**3**^**2**, generated in a nitrogen matrix, were observed to spontaneously rearrange by tunneling to 2,1-benzoisoxazole **3** and imino-ketene **4**, respectively (Scheme 2). Although the computed energy barrier for the interconversion between **s-**^**3**^**2** and **a-**^**3**^**2** is lower than for their corresponding transformation into products **3** and **4**, no conformational isomerization was observed. Therefore, these conformer-specific reactions operate simultaneously by tunneling control, giving a product ratio that cannot be rationalized by the classical reactivity models inferred by the TST.

## Results and Discussion

**Figure 1:**
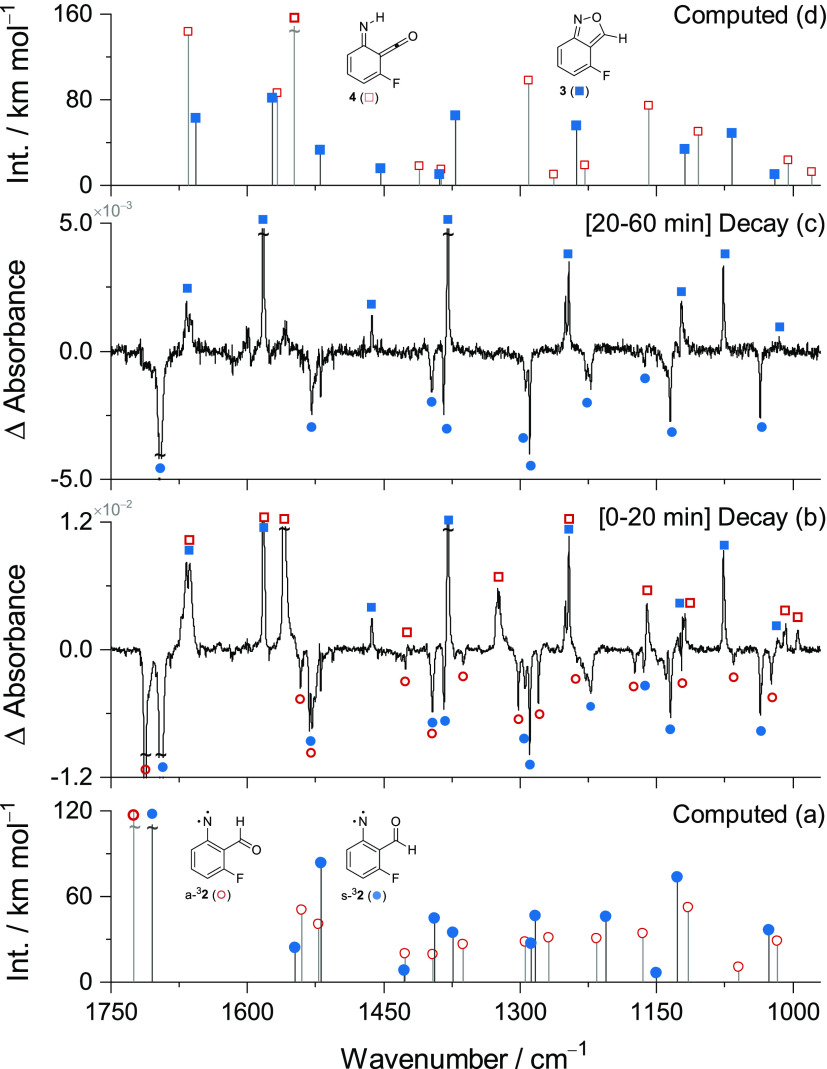
B3LYP/6-311+G(2d,p) computed IR spectra: (a) of the triplet 2-formyl-3-fluoro-phenylnitrene conformers **s-**^**3**^**2** (solid blue circles) and **a-**^**3**^**2** (open red circles) and (d) of the 2,1-benzisoxazole **3** (solid blue squares) and imino-ketene **4** (open red squares). Only computed IR transitions with intensities ≥5 km mol^–1^ are shown. Experimental difference IR spectra showing spontaneous changes of the sample (N_2_ matrix at 10 K) after the UV-irradiation of **1** (320 nm) was stopped: time interval (b) from 0 to 20 min and (c) from 20 to 60 min.

Monomers of the 2-formyl-3-fluorophenyl azide **1** precursor were isolated in a nitrogen matrix at 10 K. Experimental details are given in the Experimental and Computational Methods and in the Supporting Information (SI). The corresponding IR spectrum confirms the presence of conformers with the aldehyde in *anti* (**a-1**) and *syn* (**s-1**) orientation (relative to the azido group) in a proportion close to the computed 55:45 ratio for the gas-phase equilibrium population (Figures S1 and S2 and Table S1). The irradiation of **1** at 320 nm leads to triplet 2-formyl-3-fluorophenylnitrene ^**3**^**2** and several other products presumably from subsequent reactions (Figure S3). Interestingly, it was found that both the **a-**^**3**^**2** and the **s-**^**3**^**2** conformers were detected in a nitrogen matrix, whereas previously only the **s-**^**3**^**2** conformer was observed in argon matrix experiments.^41^ After the UV-irradiation of **1** was stopped, the simultaneous spontaneous transformations of **a-**^**3**^**2** and **s-**^3^**2** were directly probed by IR spectroscopy. Because **a-**^**3**^**2** extinguishes faster than **s-**^**3**^**2**, their spectral signatures and transformations can easily be disentangled. For instance, the difference IR spectra displayed in Figure 1 show the spontaneous consumption of both **a-**^**3**^**2** and **s-**^**3**^**2** in the first 20 min (Figure 1b) but only the spontaneous consumption of **s-**^**3**^**2** between 20 and 60 min (Figure 1c).

Some characteristic IR bands of the **s-**^**3**^**2** conformer are observed at 2886, 1697, 1396, 1294, and 1036 cm^–1^, in good agreement with the corresponding B3LYP/6-311+G(2d,p) computed IR bands at 2899 [ν(C–H)_ald._], 1705 [ν(C=O)], 1394 [δ(C–H)_ald._], 1288 [ν(CN)], and 1027 [ν(CC)_ring_, ν(C–F)] cm^–1^ (Figure 1a and 1c and Figure S4). The comprehensive assignment of the IR spectrum of **s-**^3^**2** isolated in a nitrogen matrix closely matches that reported for the compound in an argon matrix,^41^ as shown in Table S6. Representative IR bands of the **a-**^**3**^**2** conformer are observed at 2851, 1713, 1302, 1280, and 1024 cm^–1^, also in good agreement with the corresponding B3LYP/6-311+G(2d,p) computed IR bands at 2858 [ν(C–H)_ald._], 1725 [ν(C=O)], 1295 [ν(CN)], 1268 [ν(CC)_ring_, ν(C–F)], and 1017 [ν(CC)_ring_, ν(C–F)] cm^–1^ (Figure 1a and 1b and Figure S4). A detailed assignment of the IR spectrum of **a-**^**3**^**2** is given in Table S7.

Concomitantly with the consumption of **s-**^**3**^**2**, the formation of 3-fluoro-2,1-benzisoxazole **3** is observed (Figure 1c and 1d), whereas the consumption of **a-**^3^**2** results in the formation of 2-fluoro-6-imino-2,4-cyclohexadien-1-ketene **4** (Figure 1b and 1d). Some distinctive IR bands of **3** appear at ∼1665, 1582, 1379, 1123, and 1076 cm^–1^, in good agreement with the corresponding B3LYP/6-311+G(2d,p) computed IR bands at 1657 [ν(CC)], 1572 [ν(CC)], 1371 [ν(CC), δ(CH)], 1119 [ν(CO)], and 1067 [ν(C–F), δ(Is-ring)] cm^–1^. The assignment of the IR spectrum of **3** in a nitrogen matrix is in accordance with that reported for the compound in an argon matrix,^41^ as shown in detail in Table S8. Characteristic IR bands of **4** appear at 2137, ∼1668, 1559, 1160, and 1008 cm^–1^, which compare well with the corresponding B3LYP/6-311+G(2d,p) computed IR bands at 2147 [ν(C=C=O)_as_], 1665 [ν(C=C)_as_], 1549 [ν(C=C)_s_], 1159 [δ(CH)], and 1005 [ν(C–C)] cm^–1^. A comprehensive assignment of the IR spectrum of **4** is given in Table S9.

**Figure 2:**
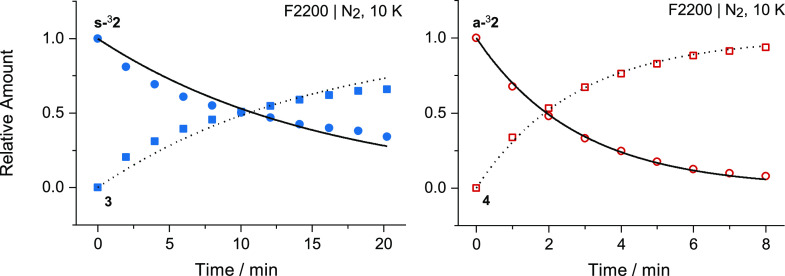
Kinetics of spontaneous rearrangement of nitrene **s-**^3^**2** to 2,1-benzisoxazole **3** (left panel) and of nitrene **a-**^3^**2** to imino-ketene **4** (right panel), in a nitrogen matrix at 10 K. The IR spectra measurements were performed using a long-pass filter transmitting only up to 2200 cm^–1^. Solid blue circles and squares represent the time evolution of the amount of **s-**^3^**2** (consumption) and **3** (production), respectively. Open red circles and squares represent the time evolution of the amount of **a-**^3^**2** (consumption) and **4** (production), respectively. The solid and dotted lines represent the best fits obtained using first-order exponential decay and growth equations, respectively (more details are given in the SI).

The kinetics of the spontaneous rearrangement of nitrene **s-**^**3**^**2** to 2,1-benzisoxazole **3** and of nitrene **a-**^**3**^**2** to imino-ketene **4**, in a nitrogen matrix at 10 K, were measured by collecting IR spectra over time using a long-pass filter blocking IR light above 2200 cm^–1^. The data were fitted with first-order exponential decay and growth equations, and the rate constants obtained were: (i) *k*_1(10 K)_ = 1.1 × 10^–3^ s^–1^ [τ_1/2_ = 10.8 min] for the consumption of **s-**^3^**2** and *k*_1_′_(10 K)_ = 1.1 × 10^–3^ s^–1^ [τ_1/2_ = 10.5 min] for the production of **3** (Figure 2, left panel);^44^ (ii) *k*_2(10 K)_ = 5.9 × 10^–3^ s^–1^ [τ_1/2_ = 1.9 min] for the consumption of **a-**^3^**2** and *k*_2_′_(10 K)_ = 6.1 × 10^–3^ s^–1^ [τ_1/2_ = 1.9 min] for the production of **4** (Figure 2, right panel). Similar rate constants were obtained when the kinetic measurements were performed by exposing the samples to the full IR radiation of the FTIR spectrometer light source (Figures S5 and S6) or by doubling the temperature to 20 K (Figures S7 and S8). Such results exclude involvement of IR-induced or thermally activated processes and provide convincing evidence for the occurrence of two tunneling reactions: (i) heavy-atom tunneling of nitrene **s-**^**3**^**2** to 2,1-benzisoxazole **3** and (ii) H-atom tunneling of nitrene **a-**^**3**^**2** to imino-ketene **4**. Note that the isomerization between **s-**^**3**^**2** and **a-**^**3**^**2** conformers can be safely excluded, as the exclusive existence of **s-**^3^**2** after ∼20 min of UV-irradiation of **1** and its subsequent consumption leading exclusively to **3** rule out any **s-**^**3**^**2 → a-**^**3**^**2** isomerization, whereas the equal rate constants for the consumption of **a-**^3^**2** and the formation of **4** rule out any **a-**^**3**^**2 → s-**^**3**^**2** isomerization. This leads to the conclusion that the two simultaneous tunneling reactions are conformer-specific and occur independently (Scheme 2).^45^

**Scheme 3:**
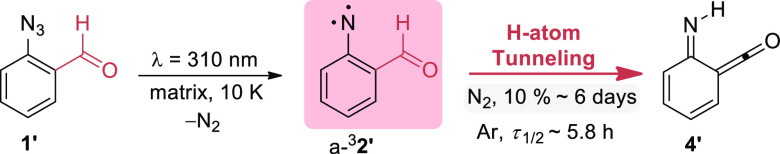
Summary of the Experimental Observations of the H-Atom Tunneling Reaction of Triplet 2-Formyl-phenylnitrene **a-**^**3**^**2′** in Nitrogen versus Argon Matrices (10 K)

Although the heavy-atom tunneling rate of **s-**^**3**^**2** to **3** in a nitrogen matrix [τ_1/2_ = 10.8 min] is similar to that reported in an argon matrix [τ_1/2_ = 8.3 min],^41^ the H-atom tunneling rate of **a-**^**3**^**2** to **4** in a nitrogen matrix [τ_1/2_ = 1.9 min] appears to be significantly slower than in experiments reported in an argon matrix, where **a-**^**3**^**2** was not detected [τ_1/2_ < few seconds]. These results indicate that the nitrogen matrix medium is crucial to make **a-**^**3**^**2** and its H-atom tunneling reaction amenable to experimental observation using steady-state spectroscopy. To support such an interpretation, we revisited the analogous H-atom tunneling reaction of triplet 2-formyl-phenylnitrene **a-**^**3**^**2′** to the corresponding imino-ketene **4′** (reported in noble-gas matrices),^42^ now using the nitrogen matrix medium (Scheme 3 and Figures S10–S12). It was found that **a-**^**3**^**2′**, generated in a nitrogen matrix, spontaneously reacts only ∼10% after 6 days (under dark conditions at 10 K), whereas the reported half-life time of **a-**^**3**^**2′** in an argon matrix is ∼5.8 h.^42^ This means that the H-atom tunneling rate of **a-**^**3**^**2′** to **4′** decreases around 2 orders of magnitude when the nitrogen matrix medium is used instead of argon.^46−49^ If a similar effect occurs for the H-atom tunneling rate of **a-**^**3**^**2** to **4**, then the extrapolated half-life time of **a-**^**3**^**2** in an argon matrix will be a few hundreds of ms (see the Kinetics section of the Experimental and Computational Methods), which explains why this species was not previously observed in the experiments carried out in argon matrices.

**Figure 3:**
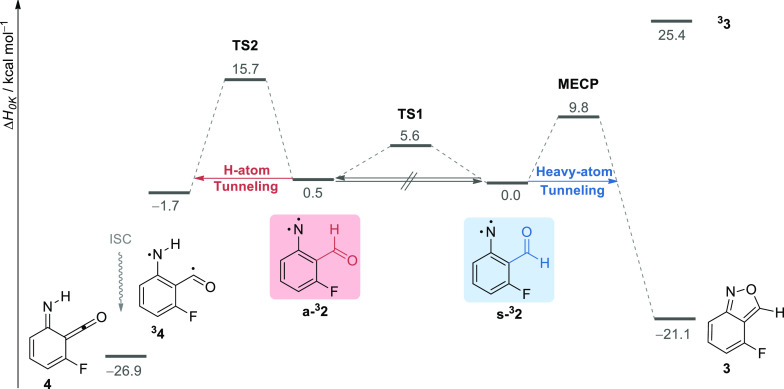
Reaction pathways for the triplet nitrene **s-**^**3**^**2** and **a-**^**3**^**2** conformers computed at the B3LYP/6-311+G(2d,p) + ZPVE level of theory. ZPVE = zero-point vibrational energy, ISC = intersystem crossing.

To acquire further knowledge on the nature of the tunneling reactions of nitrene **s-**^**3**^**2** and **a-**^**3**^**2** conformers, we performed a theoretical investigation on the relevant reaction pathways (Figure 3). The heavy-atom tunneling reaction of **s-**^**3**^**2** to **3** was previously demonstrated to occur through crossing triplet to singlet surfaces.^41,50^ Our previous computations at the M06-2X/6-311++G(d,p) level found the corresponding minimum-energy crossing point (**MECP**) at 12.1 kcal mol^–1^,^41^ whereas Heller and Richardson’s most recent computations at the MRMP(10,10)/TZVP//B2-PLYP/def2-TZVPD level found it at 10.5 kcal mol^–1^.^50^ Then, they used the state-of-the-art semiclassical golden-rule instanton theory and obtained tunneling rate constants [∼8 × 10^–3^ s^–1^; τ_1/2_ ∼ 1.5 min] in good quantitative agreement with the experiment. Here, the B3LYP/6-311+G(2d,p) **MECP** energy (or the barrier height defined as the energy difference between the **MECP** and **s-**^**3**^**2**) was found at 9.8 kcal mol^–1^, in good agreement with the value of the high-cost MRMP//B2-PLYP computations. In the case of the H-atom tunneling reaction of **a-**^**3**^**2** to **4**, computations indicate that this transformation is feasible on the triplet surface because the energy of ^**3**^**4** is lower than that of **a-**^**3**^**2**; ca. 2.2 kcal mol^–1^ at the B3LYP level or 2.6 kcal mol^–1^ at the highly accurate CBS-APNO method (see also Table S11). The B3LYP computed energy barrier of **a-**^**3**^**2** to ^**3**^**4** (**TS2**) is 15.2 kcal mol^–1^ (all values are relative to the **a-**^**3**^**2** energy), in the same range of the CBS-APNO value of 15.2 kcal mol^–1^ (whereas M06-2X also overestimated this barrier with a value of 21.0 kcal mol^–1^; see Table S11). Applying the Wentzel–Kramers–Brillouin (WKB) model on the B3LYP-computed reaction path [height = 15.2 kcal mol^–1^ and width = 1.09 Å; Figure S13], an H-atom tunneling rate of 2.5 s^–1^ [τ_1/2_ ∼ 0.3 s] is estimated for the reaction of **a-**^**3**^**2** to ^**3**^**4** (details are given in the Kinetics section of the Experimental and Computational Methods), which is in good agreement with the experimental observations, particularly considering the extrapolation rate presented for the more inert argon matrix medium. Applying the same theoretical approach, a slower H-atom tunneling rate of 9.3 × 10^–4^ s^–1^ [τ_1/2_ = 12.4 min] is estimated for the reaction of **a-**^**3**^**2′** to ^**3**^**4′** [height = 17.3 kcal mol^–1^ and width = 1.29 Å; Figure S14], also in reasonable agreement with the experimental observations. A connection between triplet **a-**^**3**^**2** and singlet **4** via an MECP was not found. Thus, the computed data provide compelling evidence that the reaction of **a-**^**3**^**2** to **4** occurs by a mechanism involving H-atom tunneling on the corresponding triplet surface followed by intersystem crossing (Figure 3).^51^

Remarkably, computations also show that the exclusive transformations of **s-**^**3**^**2** to **3** and of **a-**^**3**^**2** to **4** independently occur through significantly higher energy barriers than the possible competitive conformational isomerization of **a-**^**3**^**2** to **s-**^**3**^**2** (not observed), which is estimated to be only ∼5 kcal mol^–1^ (**TS1**). Therefore, we reveal that the simultaneously observed conformer-specific reactions of triplet 2-formyl-3-fluorophenylnitrene ^**3**^**2** operate through an intriguing manifestation of tunneling control, and the classical Curtin–Hammett principle does not apply. One might wonder why the isomerization of **a-**^**3**^**2** to **s-**^**3**^**2** does not occur by a tunneling process. Although the corresponding reaction barrier has a height of only ∼5 kcal mol^–1^, the B3LYP-computed reaction path also shows that it has a significant width of ∼3.32 Å (Figure S15). Applying the WKB model, it is therefore evident that not even a light H-atom can efficiently penetrate through such a barrier, as a hypothetical H-atom tunneling would imply a rate of 8.2 × 10^–12^ s^–1^ [τ_1/2_ ∼ 8.5 × 10^10^ s] (details are given in the Kinetics section of the Experimental and Computational Methods).^52^

## Conclusions

We have demonstrated here an unprecedented example where two conformers of the same species react simultaneously and independently by pure quantum tunneling to distinct rearrangement products, against the reactivity predictions inferred by TST. The *syn* and *anti* aldehyde conformers of triplet 2-formyl-3-fluorophenylnitrene (**s-**^**3**^**2** and **a-**^**3**^**2**), generated in a nitrogen matrix by UV-irradiation of azide precursor **1**, were found to spontaneously rearrange to the corresponding 2,1-benzisoxazole **3** and imino-ketene **4**, respectively. The kinetics of these transformations were measured by IR spectroscopy, and temperature-independent rate constants of ∼1 × 10^–3^ s^–1^ [τ_1/2_ ∼ 11 min] (**s-**^**3**^**2 → 3**) and ∼6 × 10^–3^ s^–1^ [τ_1/2_ ∼ 2 min] (**a-**^**3**^**2 → 4**) were obtained in nitrogen matrices at 10 and 20 K. The occurrence of conformational isomerization between **s-**^**3**^**2** and **a-**^**3**^**2** was not observed, and the existence of any IR-induced process was excluded. This provides unequivocal evidence for the occurrence of heavy-atom tunneling of **s-**^**3**^**2** to **3** and of H-atom tunneling of **a-**^**3**^**2** to **4**. Computations confirm the existence of such tunneling reaction pathways and show that they have significantly higher energy barriers than the *a priori* competitive conformational isomerization between **s-**^**3**^**2** and **a-**^**3**^**2**. Therefore, a remarkable manifestation of simultaneous tunneling control operates on the singular conformer-specific tunneling reactions of **s-**^**3**^**2** and **a-**^**3**^**2**, yielding a product outcome that cannot be rationalized by the conventional kinetic or thermodynamic control. This fascinating tunneling-driven example constitutes an important discovery to better understand the chemical reactivity governed by quantum tunneling, also highlighting the limitations of the current classical theories.

## Experimental and Computational Methods

### Samples

The 2-formyl-3-fluorophenyl azide **1** and the 2-formyl-phenyl azide **1′** were prepared as reported in our previous publications: see refs (41 and 42).

### Matrix Isolation Spectroscopy

A sample of **1** or **1′** was placed in a glass tube which was connected to the vacuum chamber of a cryostat through a stainless-steel needle valve. Prior to the experiments, volatile impurities were removed from the sample by pumping through the cryostat at room temperature. The matrices were then prepared by codeposition of sample vapors at room temperature along with an excess of nitrogen gas (N50, Air–Liquid) onto the optical substrate at 15 K. A CsI window used as the optical substrate was cooled to 15 K using a closed-cycle helium cryostat (APD Cryogenics HC-2 compressor with a DE-202A expander). The temperature of the cold window was measured directly by a silicon diode sensor connected to a digital controller (LakeShore 331), providing stabilization with an accuracy of 0.1 K. The temperature was changed to 10 or 20 K after deposition and kept at that temperature during the irradiation experiments and during the monitoring of spontaneous transformations.

A Thermo Nicolet 6700 Fourier transform infrared spectrometer, equipped with a liquid nitrogen cooled mercury cadmium telluride (MCT-B) detector and a KBr beam splitter, was employed to record the mid-IR spectra (4000–400 cm^–1^ range) with 0.5 cm^–1^ resolution. In some experiments, to avoid exposure of the matrix sample to the full broad-band radiation of the FTIR spectrometer source (ETC EverGlo globar, which provides energy within the 7400–50 cm^–1^ range), a long-pass filter was used to block the IR light with wavenumbers above ∼2200 cm^–1^ (Edmund Optics). A stream of dry air was continually purged through the optical path of the spectrometer to avoid interference from atmospheric H_2_O and CO_2_.

### UV–vis Irradiation Experiments

The matrices were irradiated through the outer KBr window of the cryostat, using tunable narrow-band light with a full width at half-maximum (fwhm) of ∼0.2 cm^–1^, provided by a signal (visible light) or a frequency-doubled signal (UV range) beam of an optical parametric oscillator (Spectra Physics Quanta-Ray MOPO-SL) pumped with a pulsed Nd:YAG laser (Spectra-Physics PRO-230: output power ≈4.3 W; wavelength = 355 nm; duration = 10 ns; repetition rate = 10 Hz).

### Kinetics

The kinetics measurements of the spontaneous transformation in nitrogen matrices of nitrene **s-**^**3**^**2** to 2,1-benzisoxazole **3** and of nitrene **a-**^**3**^**2** to imino-ketene **4** were first carried out using an Edmund Optics long-pass filter to block the IR light with ν > 2200 cm^–1^ from the spectrometer FTIR globar source. The data collection was carried out at 1 min intervals by acquiring mid-IR spectra in the 2200–400 cm^–1^ range with 35 scans (collection length = 58 s). This procedure was followed at two different temperatures, 10 and 20 K. Then, to examine any effect of the IR radiation from the globar source of the FTIR spectrometer, the same kinetics measurements were carried out but without using any long-pass filter and acquiring mid-IR spectra in the 4000–400 cm^–1^ range. In all cases, the moment of recording the first spectrum (a few seconds after the irradiation of **1** stopped) was assumed to be the origin of the kinetics analysis (time = 0 min). Therefore, in the first spectrum, the reactant IR band at 1289 cm^–1^ for **s-**^**3**^**2** and at 1712 cm^–1^ for **a-**^**3**^**2** was assumed to be 100%, and the product IR band at 1379 cm^–1^ for **3** and at 1559 cm^–1^ for **4** was assumed to be 0%. The selection of the mentioned IR bands was based on their high intensity and nonoverlapping absorption. Then, the relative amount of each species was monitored as a function of time by following the intensity of those IR bands. Finally, first-order kinetic exponential decay and growth equations were fitted to the experimental data, and the kinetic constants and the corresponding half-lives were obtained as discussed in the text.

A rough extrapolation for the rate constant of **a-**^**3**^**2** to **4** in an argon matrix: (i) Considering the measured 10% consumption of **a-**^**3**^**2′** to **4′** in a nitrogen matrix at 10 K at the end of 6 days, this gives approximately a rate constant of 2.0 × 10^–7^ s^–1^. (ii) Considering the measured 50% consumption of **a-**^**3**^**2′** to **4′** in an argon matrix at 10 K at the end of 5.8 h, this gives approximately a rate constant of 3.3 × 10^–5^ s^–1^. (iii) Therefore, the rate constant for the reaction of **a-**^**3**^**2′** to **4′** is ∼165 times slower in a nitrogen matrix than in an argon matrix. (iv) The measured rate constant of **a-**^**3**^**2** to **4** in a nitrogen matrix at 10 K was 6.1 × 10^–3^ s^–1^ [τ_1/2_ = 1.9 min]. (v) Therefore, assuming that the rate constant of **a-**^**3**^**2** to **4** is 165 times slower in a nitrogen matrix than in an argon matrix, the extrapolated rate constant of **a-**^**3**^**2** to **4** in an argon matrix is 1.0 s^–1^ [τ_1/2_ = 0.7 s].

### IR Spectrum Computations

To support the assignment of the experimental IR spectra, geometry optimizations and harmonic vibrational frequencies were computed at the B3LYP/6-311+G(2d,p)^53−55^ level of theory, using Gaussian 16 (Revision B.01).^56^ The corresponding Hessian matrix was inspected to confirm the nature of each stationary point. To compensate the neglected anharmonic effects, incomplete treatment of electron correlation, and basis set limitations, the harmonic vibrational frequencies were scaled by a factor of 0.979.^25^ Some of the simulated spectra were prepared by convolute with Lorentzian functions (full width at half-maximum = 2 cm^–1^) the resulting frequencies together with the IR intensities. In such cases, the integral band intensities correspond to the calculated IR absolute intensities and are presented in the arbitrary units of “Relative Intensity”.

### Normal Mode Analysis

The theoretical normal modes of **s-**^**3**^**2**, **a-**^**3**^**2**, **3**, **4**, and **5** were analyzed by performing the potential energy distribution (PED) calculations. The calculated force constants with respect to Cartesian coordinates were transformed into the force constants with respect to internal coordinates, which allowed the PED analysis to be carried out as described by Schachtschneider and Mortimer.^57^ The set of internal coordinates for **s-**^**3**^**2**/**a-**^**3**^**2**, **3**, **4**, and **5** were defined as recommended by Pulay et al.^58^ and are given in Tables S2–S5, respectively. The atom numberings of **s-**^**3**^**2**/**a-**^**3**^**2**, **3**, **4**, and **5**, used for the internal coordinate definition purposes, are presented in Figure S16. The resulting vibrational assignments are presented in Tables S6–S10.

### Computations of the PES

All calculations regarding the relevant potential energy surface (PES) for the reaction of the triplet nitrene **s-**^**3**^**2** and **a-**^**3**^**2** conformers were performed using Gaussian 16 (Revision B.01).^56^ The geometry optimizations and harmonic frequencies of the minima and the first-order transition states were computed at the M06-2X/6-311++G(d,p),^59^ B3LYP/6-311+G(2d,p),^53−55^ and CBS-APNO^60^ levels of theory.

The M06-2X/6-311++G(d,p) level was previously employed to compute the crossing of triplet and singlet surfaces of nitrene **s-**^**3**^**2** and 2,1-benzisoxazole **3**.^41^ The part of the PES connecting **s-**^**3**^**2** to **a-**^**3**^**2** and **a-**^**3**^**2** to **4** was computed here for the first time. For these computations, the M06-2X/6-311++G(d,p) method was also employed. The entire PES addressed here was also computed at the B3LYP/6-311+G(2d,p) level. The B3LYP minimum energy crossing point (MECP) connecting **s-**^**3**^**2** and **3** was found using a global optimization algorithm employed in the EasyMECP software package^61^ (the M06-2X/6-311++G(d,p) MECP geometry was used as an initial guess). A search for a MECP connecting **a-**^**3**^**2** and **4** was also performed to investigate the possibility of the corresponding tunneling reaction that occurs through crossing potential energy surfaces. Initially, a partial optimization method^62^ was employed by running relaxed scans as a function of either *r*(N7H10) or *r*(C8H10), starting from the optimized structures of **a-**^**3**^**2**, **4**, and ^**3**^**4**. No crossing surfaces were found. This could be because neither of the selected internal coordinates resembles well the expected reaction coordinate on the triplet or singlet surfaces. Then, a global optimization search was tried. Twenty-one geometries spaced by 0.2 bohr along the IRC connecting **a-**^**3**^**2** and ^**3**^**4** were selected as initial guesses for the search for a MECP using the EasyMECP algorithm. However, no MECP connecting **a-**^**3**^**2** and **4** was found.

The part of the PES connecting **s-**^**3**^**2** to **a-**^**3**^**2** and **a-**^**3**^**2** to ^**3**^**4** was also computed using the highly accurate CBS-APNO method. The CBS-APNO energies were found to be in better agreement with those obtained at the B3LYP level than at the M06-2X level. Therefore, the B3LYP/6-311+G(2d,p) level was employed to compute the corresponding potential energy profiles, using the intrinsic reaction coordinate (IRC), for the tunneling rate calculations, as described in the following section.

### Tunneling Rates

Tunneling computations were performed on the B3LYP/6-311+G(2d,p) computed potential energy profiles using the intrinsic reaction coordinate (IRC) in non-mass-weighted Cartesian coordinates.^17^ The transmission coefficients for H-atom tunneling through such barriers were computed using the Wentzel–Kramers–Brillouin (WKB) approximation.^63−65^ Hence, the probability *P*(*E*) of tunneling is given by eq 1:^66^
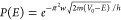
1where a particle with mass *m* tunnels through a barrier with height *V*_0_ and width *w*; (*V*_0_ – *E*) is the energy deficiency of the particle with respect to the top of the barrier; and *h* is Planck’s constant.

(i) For the reaction of nitrene **a-**^**3**^**2** to imino-ketene ^**3**^**4**, the computed probability of tunneling was estimated to be equal to 5.9 × 10^–14^, using the calculated barrier height of 63.8 kJ mol^–1^ (15.2 kcal mol^–1^) and width at the ZPVE level of 2.06 bohr (1.09 Å) (Figure S13). The tunneling rate is the product of the probability of tunneling (transmission coefficient) and the frequency of attempts. If the H-atom of the CHO moiety of **a-**^**3**^**2** is vibrating at a δ(CH) frequency of about 1396 cm^–1^ [B3LYP/6-311+G(2d,p) scaled computed value], this results in a tunneling rate of 2.6 s^–1^, i.e., a half-life time of 2.8 × 10^–1^ s.
(ii) For the reaction of nitrene **a-**^**3**^**2′** to imino-ketene ^**3**^**4′**, the computed probability of tunneling was estimated to be equal to 2.3 × 10^–17^, using the calculated barrier height of 72.6 kJ mol^–1^ (17.3 kcal mol^–1^) and width at the ZPVE level of 2.43 bohr (1.29 Å) (Figure S14). If the H-atom of the CHO moiety of **a-**^**3**^**2′** is vibrating at a δ(CH) frequency of about 1370 cm^–1^ [B3LYP/6-311+G(2d,p) scaled computed value], this results in a tunneling rate of 9.3 × 10^–4^ s^–1^, i.e., a half-life time of 7.5 × 10^2^ s.
(iii) For the sake of the argument regarding the impossibility of conformational isomerization tunneling between **a-**^**3**^**2** and **s-**^**3**^**2**, we assumed a hypothetical H-atom penetration through that barrier. The computed probability of tunneling was estimated to be equal to 3.7 × 10^–24^, using the calculated barrier height of 21.6 kJ mol^–1^ (5.2 kcal mol^–1^) and width at the ZPVE level of 6.27 bohr (3.32 Å) (Figure S15). Considering the CHO moiety of **a-**^**3**^**2** vibrating at a τ(CHO) frequency of about 74 cm^–1^ [B3LYP/6-311+G(2d,p) scaled computed value], this results in a tunneling rate of 8.2 × 10^–12^ s^–1^, i.e., a half-life time of 8.5 × 10^10^ s. It must be highlighted that, in fact, the conformational isomerization of **a-**^**3**^**2** to **s-**^**3**^**2** involves the movement of a much heavier CHO fragment, which makes the occurrence of tunneling even much less effective than that estimated considering only the movement of an H-atom.

## Supporting Information Available

Additional experimental and theoretical results, vibrational assignments, and computational data are provided (PDF)
