# Supplementary material for: Simultaneous Tunneling Control in Conformer-Specific Reactions
Source: J Am Chem Soc. 2022 Nov 2;144(45):20866–74. doi: 10.1021/jacs.2c09026 (PMC9776521; doi:10.1021/jacs.2c09026)
Supplement: Supplementary file 1 — ja2c09026_si_001.pdf [file ja2c09026_si_001.pdf]

Supporting Information

Simultaneous Tunneling Control in Conformer-Specific Reactions

Cláudio M. Nunes,[1]* José P. L. Roque,[1] Srinivas Doddipatla,[1] Samuel A. Wood,[2] Robert J. McMahon,[2] and Rui Fausto[1]

[1]University of Coimbra, CQC-IMS, Department of Chemistry, 3004-535 Coimbra, Portugal
[2]Department of Chemistry, University of Wisconsin-Madison, Wisconsin 53706-1322, United States

TABLE OF CONTENTS

1. Figures **S2**

2. Tables **S18**

3. Computational Data **S29**

4. References **S40**

1. Figures

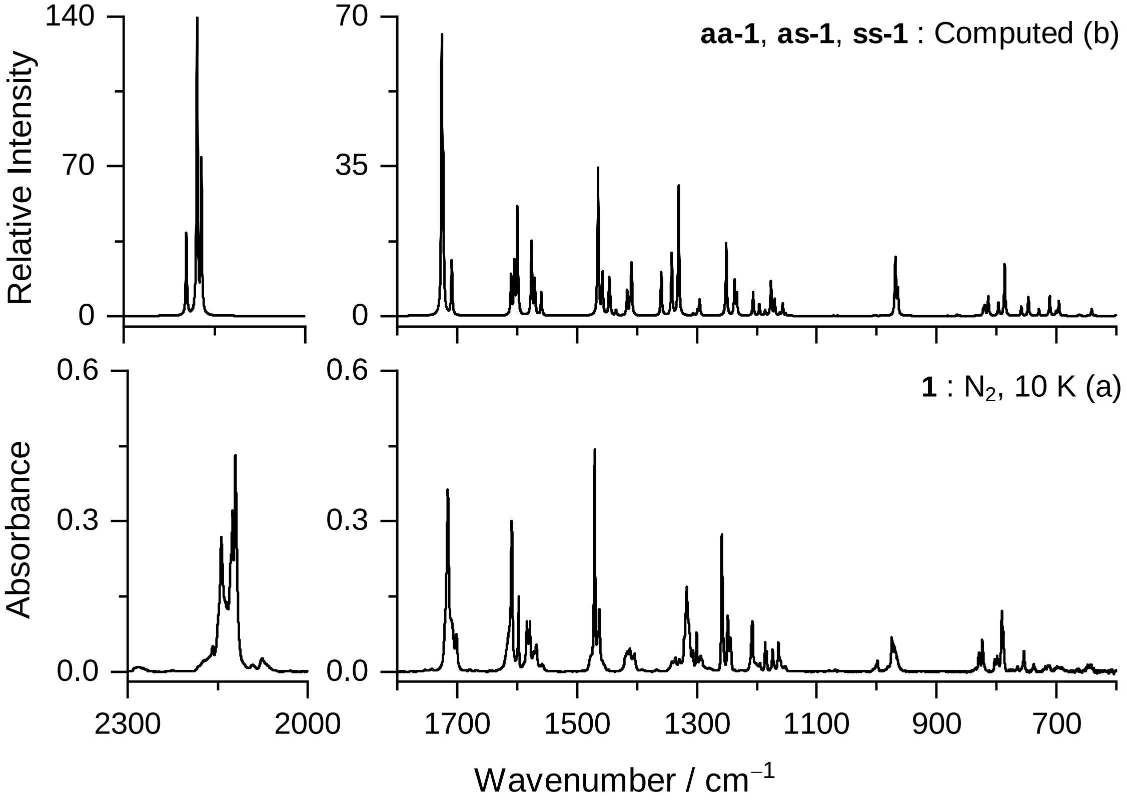

Figure S1. (a) Experimental IR spectrum of 2-formyl-3-fluorophenylazide **1** isolated in a nitrogen matrix at 10 K. (b) B3LYP/6-311+G(2d,p) computed IR spectrum of **1** considering the population of conformers **aa-1**, **as-1** and **ss-1** in a ratio of 0.55:0.26:0.19 (see Table S1). Note that in the **aa-1** conformer the aldehyde adopts *anti* orientation (simplified notation mentioned in the text is **a-1**), whereas in the **as-1** and **ss-1** conformers the aldehyde adopts *syn* orientation (simplified notation mentioned in the text is **s-1**).

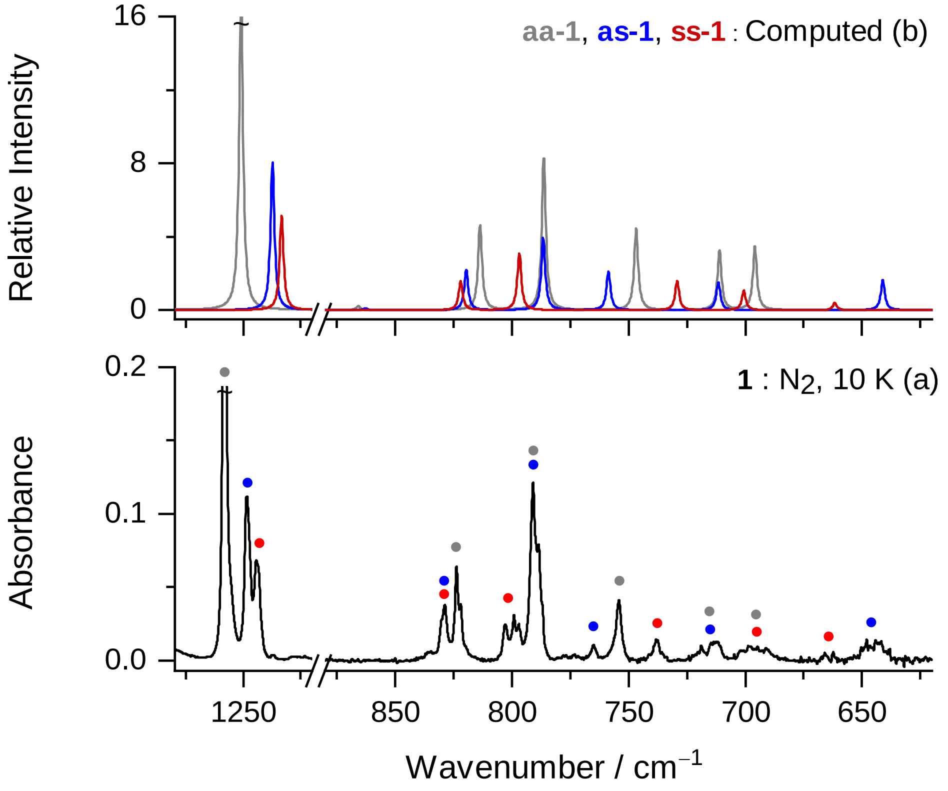

Figure S2. (a) Selected fingerprint region of the experimental IR spectrum of 2-formyl-3-fluorophenylazide **1** isolated in a nitrogen matrix at 10 K. Bands assigned to **aa-1**, **as-1** and **ss-1** conformers are indicated by solid circles with gray, blue, and red color, respectively. (b) IR spectrum of **aa-1** (gray), **as-1** (blue), and **ss-1** (red) computed at the B3LYP/6-311+G(2d,p) level of theory considering the population of conformers in a ratio of 0.55:0.26:0.19, respectively. Note that in the **aa-1** conformer the aldehyde adopts *anti* orientation (simplified notation mentioned in the text is **a-1**), whereas in the **as-1** and **ss-1** conformers the aldehyde adopts *syn* orientation (simplified notation mentioned in the text is **s-1**).

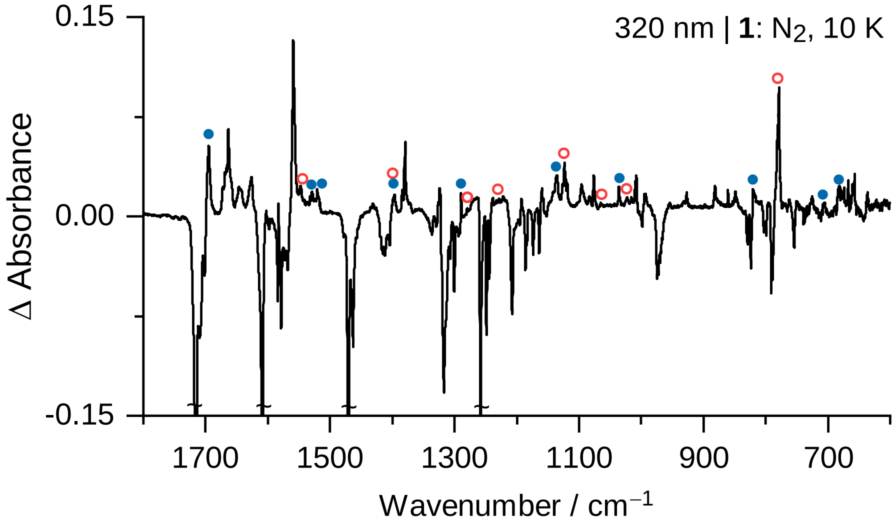

Figure S3. Experimental difference IR spectrum showing changes after irradiation at λ = 320 nm (20 s, 20 mW) of 2-formyl-3-fluorophenylazide **1** isolated in a nitrogen matrix at 10 K. The downward bands are due to the consumed **1**, and the upward bands are due to the photoproduced species assigned to **s-^{3}2** (●, solid blue circles) and **a-^{3}2** (○, open red circles). The generation of products **3**, **4**, and **5** were also detected after the irradiation but the corresponding upward bands were not signed to avoid congestion. The unambiguous assignment of each product is discussed further in the text.

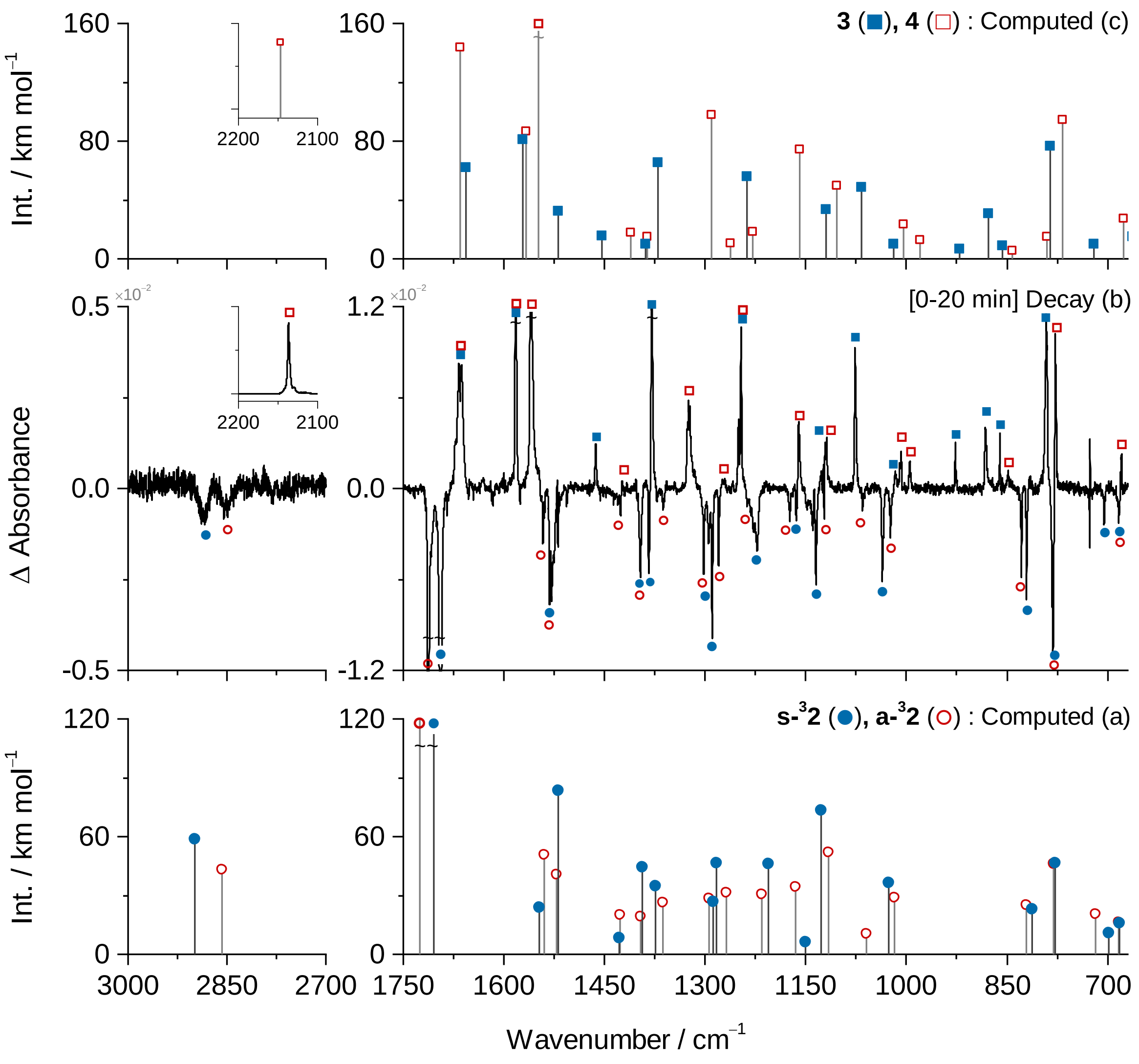

Figure S4. B3LYP/6-311+G(2d,p) computed IR spectra: (a) of the triplet 2-formyl-3-fluoro-phenylnitrene conformers **s-^{3}2** (●, solid blue circles) and **a-^{3}2** (○, open red circles); and (c) of the 2,1-benzisoxazole **3** (■, solid blue squares) and imino-ketene **4** (□, open red squares). Only computed IR transitions with intensities ≥ 5 km mol^{-1} are shown. (b) Experimental difference IR spectrum showing spontaneous changes of the sample (N_2 matrix at 10 K) at 20 min after the irradiation of **1** (320 nm) was stopped.

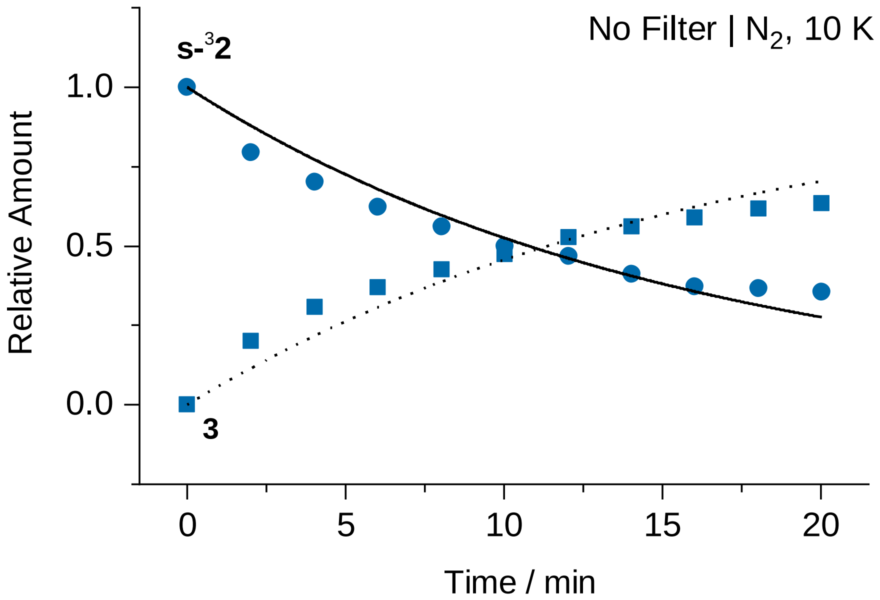

Figure S5. Kinetics of spontaneous rearrangement of nitrene **s-**^{3}**2** to 2,1-benzisoxazole **3** in a nitrogen matrix at 10 K. The sample was exposed to the full IR radiation of the spectrometer source. Solid blue circles (●) and squares (■) represent the time evolution of the amount of **s-**^{3}**2** (consumption) and **3** (production), respectively. The solid and dotted line represent the best fits obtained using a first-order exponential decay and growth equations, respectively. The rate constant obtained were $k_{1(10\ K)} = 1.1 \times 10^{-3}\ s^{-1}$ ($\tau_{1/2} = 10.8$ min) for the consumption of **s-**^{3}**2** and $k_1'{}_{(10\ K)} = 1.0 \times 10^{-3}\ s^{-1}$ ($\tau_{1/2} = 11.4$ min) for the production of **3**.

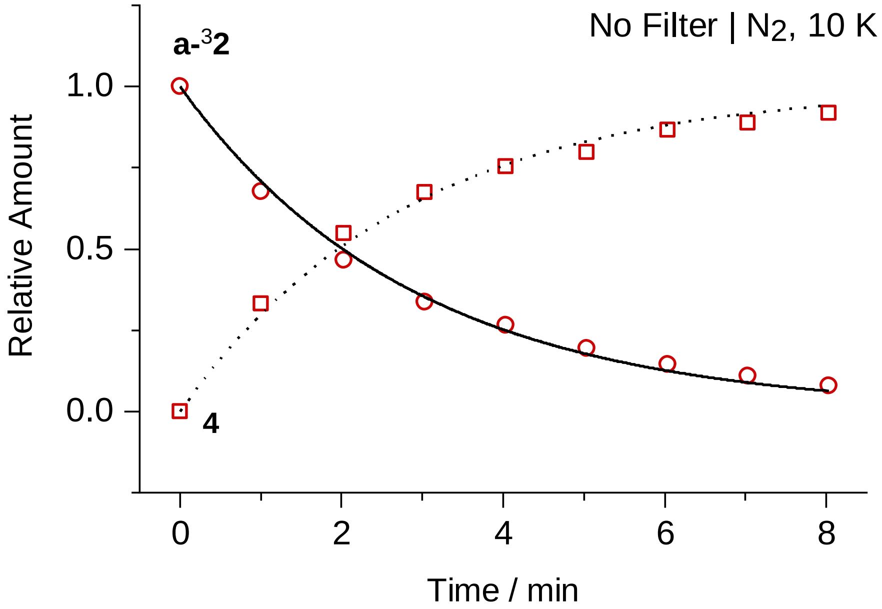

Figure S6. Kinetics of spontaneous rearrangement of nitrene **a-**^{3}**2** to imine-ketene **4** in a nitrogen matrix at 10 K. The sample was exposed to the full IR radiation of the spectrometer source. Open red circles (○) and squares (□) represent the time evolution of the amount of **a-**^{3}**2** (consumption) and **4** (production), respectively. The solid and dotted line represent the best fits obtained using a first-order exponential decay and growth equations, respectively. The rate constant obtained were $k_{2(10\ K)} = 5.7 \times 10^{-3}$ s^{-1} ($\tau_{1/2} = 2.0$ min) for the consumption of **a-**^{3}**2** and $k_2'_{(10\ K)} = 5.9 \times 10^{-3}$ s^{-1} ($\tau_{1/2} = 2.0$ min) for the production of **4**.

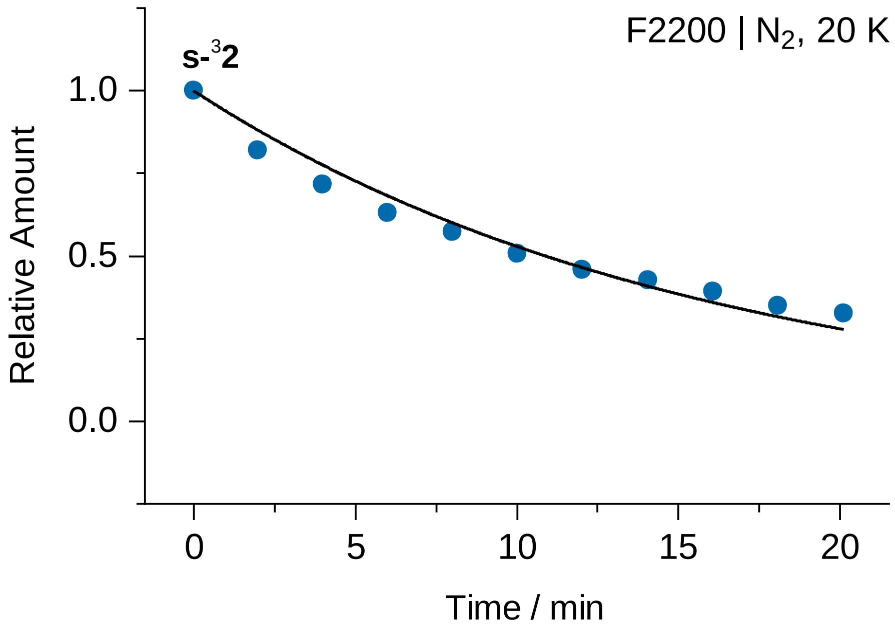

Figure S7. Kinetics of spontaneous rearrangement of nitrene **s-**^{3}**2** to 2,1-benzisoxazole **3** in a nitrogen matrix at 20 K. The IR spectra measurements were performed using a longpass filter transmitting only up 2200 cm^{-1}. Solid blue circles (●) represent the time evolution of the amount of **s-**^{3}**2** (consumption), and the solid line represent the best fit obtained using a first-order exponential decay equation. The rate constant obtained were $k_{1(20\ K)} = 1.1 \times 10^{-3}\ s^{-1}$ ($\tau_{1/2} = 10.9$ min) for the consumption of **s-**^{3}**2**.

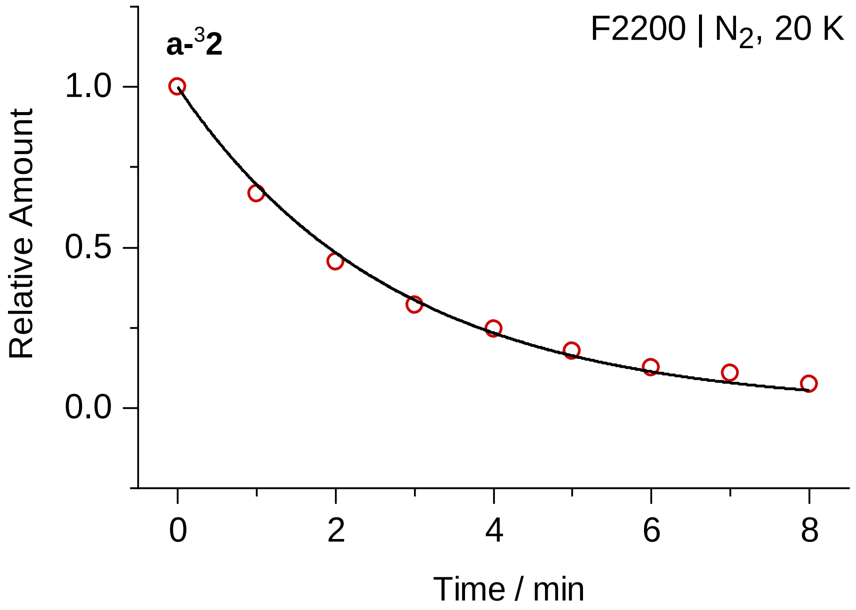

Figure S8. Kinetics of spontaneous rearrangement of nitrene **a-**^{3}**2** to imino-ketene **4** in a nitrogen matrix at 20 K. The IR spectra measurements were performed using a longpass filter transmitting only up 2200 cm^{-1}. Open red circles (o) represent the time evolution of the amount of **a-**^{3}**2** (consumption), and the solid line represent the best fits obtained using a first-order exponential decay equation. The rate constant obtained was $k_{2(20\ K)}$ = 6.1 × 10^{-3} s^{-1} ($\tau_{1/2}$ = 1.9 min) for the consumption of **a-**^{3}**2**.

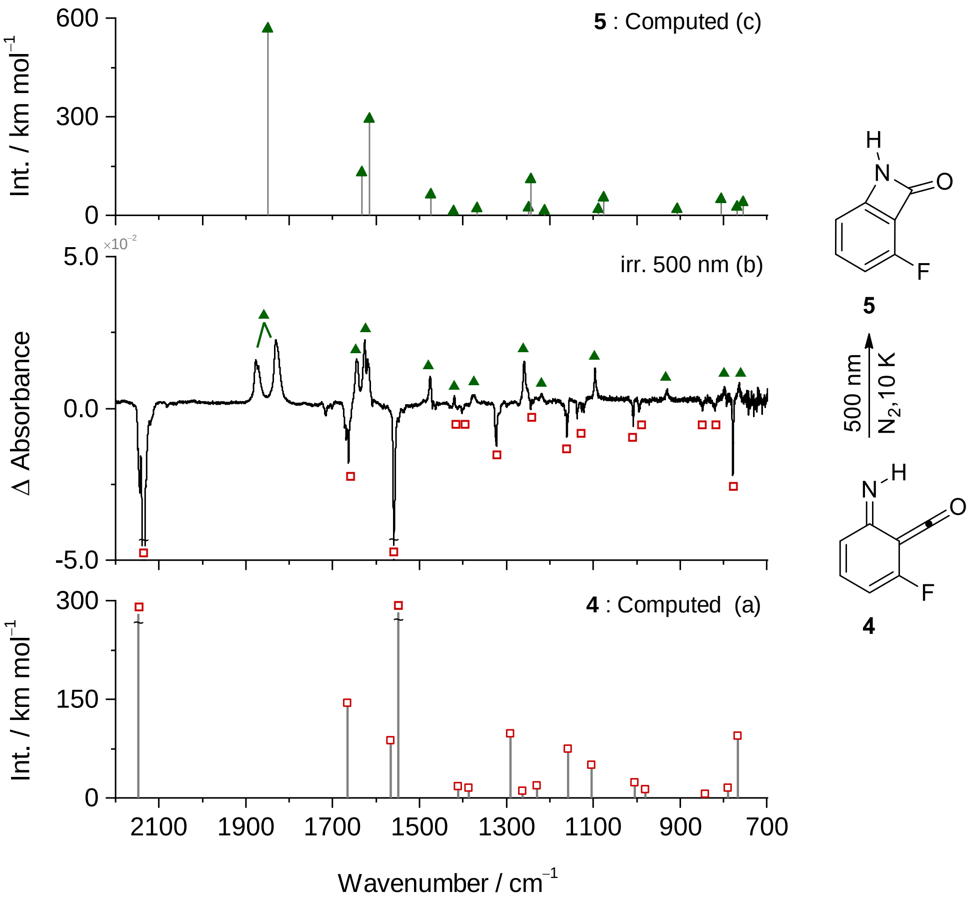

Figure S9. B3LYP/6-311+G(2d,p) computed IR spectrum of (a) imino-ketene **4** (□, open red squares) and (c) benzoazetinone **5** (▲, solid green triangles). Only computed IR transitions with intensities ≥ 5 km mol^{-1} are shown. (b) Experimental difference IR spectrum showing changes after visible light irradiation (λ = 500 nm; 25 min; 25 mW) subsequently to the spontaneous transformation of nitrene conformers **a-^{3}2** and **s-^{3}2**, which were generated by UV-irradiation of **1** in a nitrogen matrix at 10 K. The downward bands are due to the consumed **4** and the upward bands are due to the produced **5**. The photochemical transformation of an imino-ketene into a benzoazetinone is not unprecedented: see ref. S1 and references cited therein.

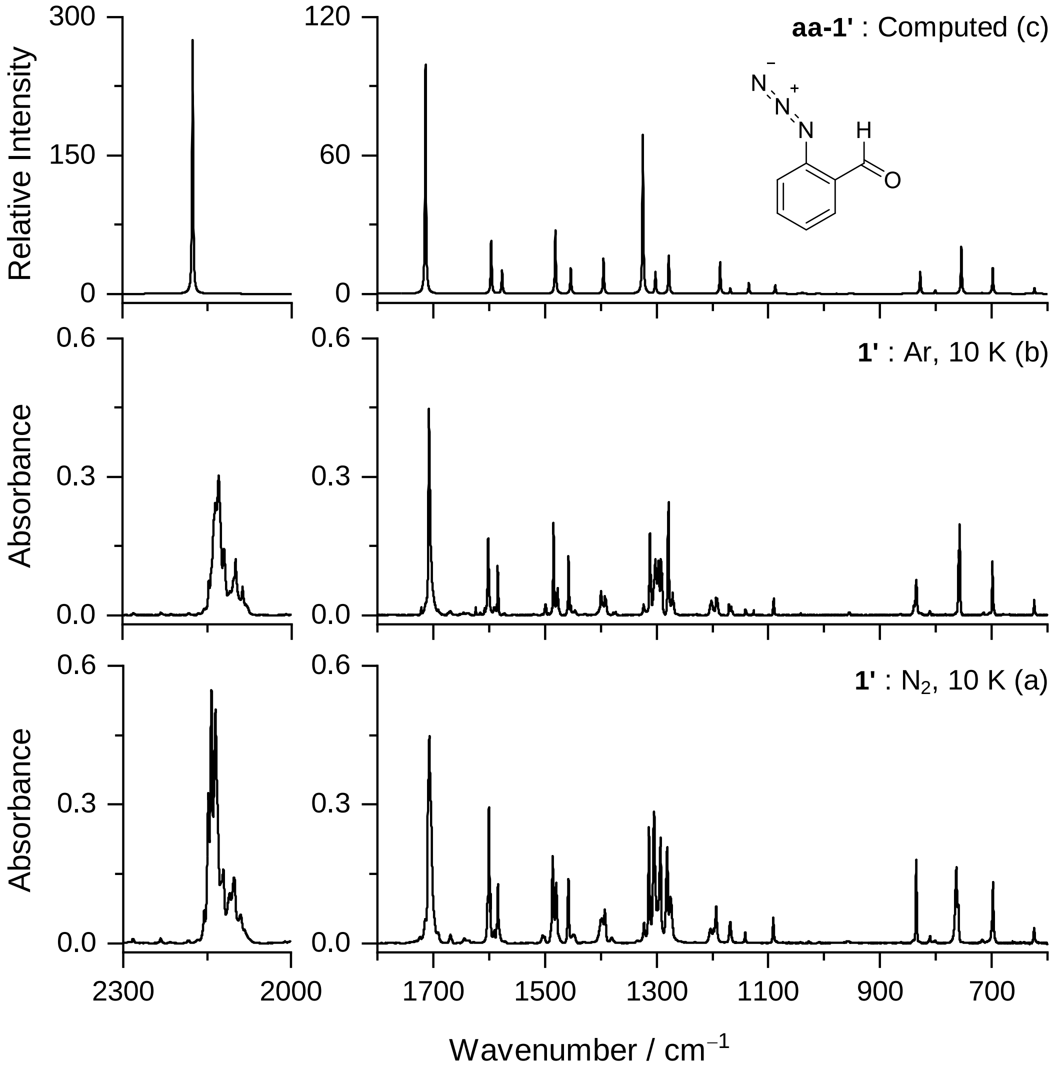

Figure S10. Experimental IR spectrum of 2-formylphenylazide **1'** isolated (a) in a nitrogen and (b) in an argon matrix at 10 K (ref. S2). (c) B3LYP/6-311+G(2d,p) computed IR spectrum of azide **aa-1'** conformer Note that only the most stable azide **aa-1'** conformer is isolated in the matrix isolation experiments. The other three possible conformers (**as-1'**, **as-1'**, and **ss-1'**) are at least 10 kJ mol^{-1} more energetic and, therefore, have negligible populations at room temperature in the equilibrium gas-phase prior to deposition. In accordance, the experimental IR spectrum of matrix-isolated **1'** shows only a good agreement with the calculated IR spectrum of conformer **aa-1'**. More details are reported in ref. S2.

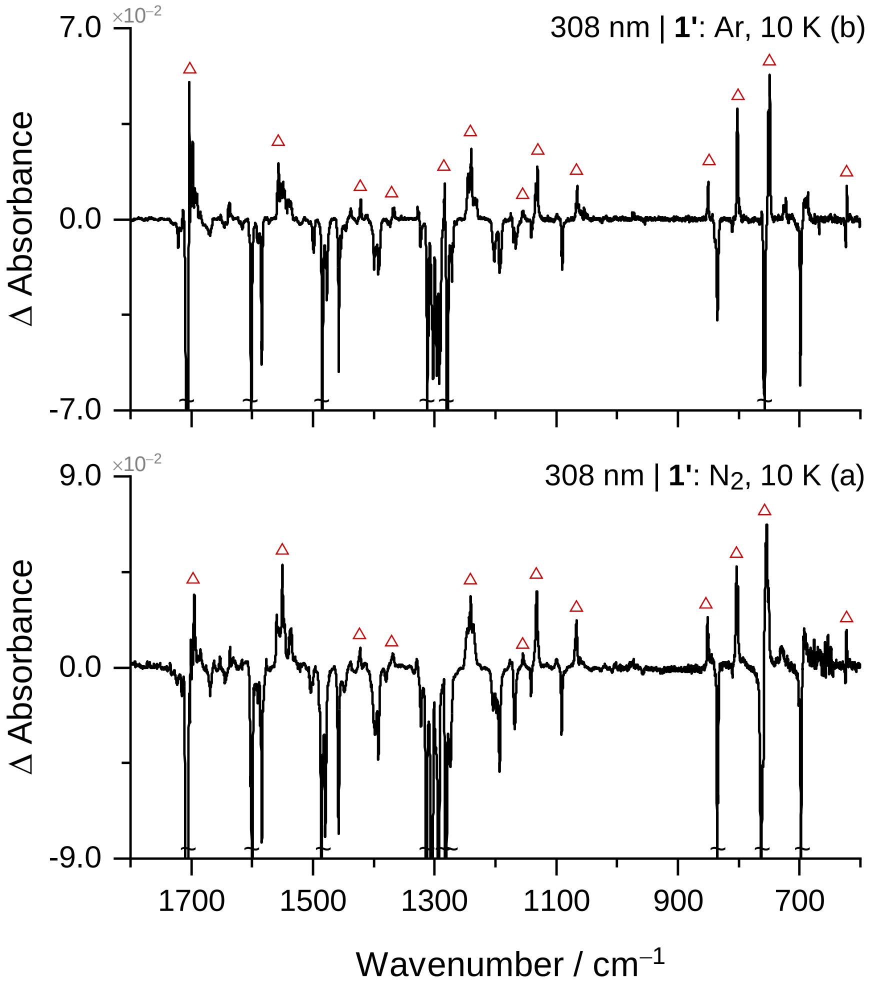

Figure S11. (a) Experimental difference IR spectrum showing changes after irradiation at λ = 308 nm (15 min, 8 mW) of 2-formylphenylazide **1'** isolated in a nitrogen matrix at 10 K. (b) Experimental difference IR spectrum showing the formation of triplet nitrene **a-^{3}2'** upon irradiation of azide **1'** (λ = 308 nm) in an argon matrix at 10 K, as reported in ref. S2. The downward bands are due to the consumed **1'**, and the signed upward bands (△, open red triangles) are due to the photoproduced species assigned to nitrene **a-^{3}2'**.

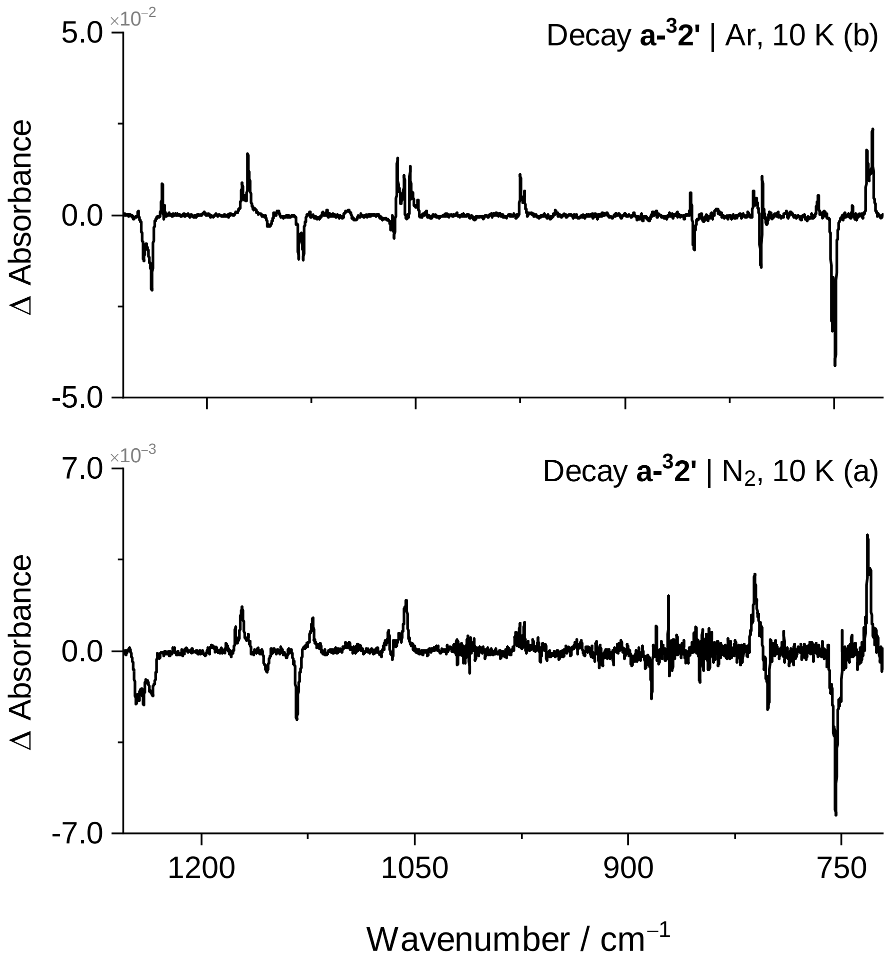

Figure S12. (a) Experimental difference IR spectrum after keeping the sample for ~6 days under dark conditions, upon UV-irradiation (λ = 308 nm) of 2-formylphenylazide **1'** isolated in a nitrogen matrix at 10 K. (b) Experimental difference IR spectrum after keeping the sample for 27.5 h under dark conditions, upon UV-irradiation (λ = 308 nm) of **1'** isolated in Ar matrix at 10 K, as reported in ref. S2. The downward bands are due to the triple nitrene **a-^{3}2'** and the upward bands due to imino-ketene **4'**, which identify the H-atom tunneling reaction of **a-^{3}2'** to **4'**, as reported in ref. S2.

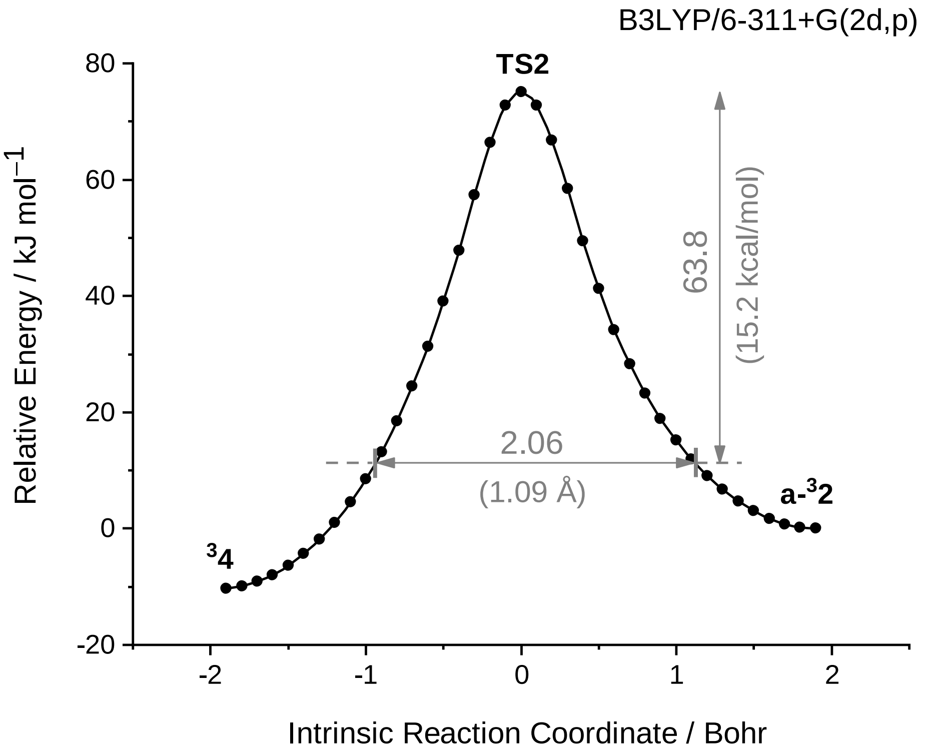

Figure S13. Relative electronic energy as a function of intrinsic reaction coordinate (IRC) for the H-shift reaction from **a-^{3}2** to **^{3}4** computed at the B3LYP/6-311+G(2d,p) level in non-mass-weighted (Cartesian) coordinates. The vertical arrow designates the B3LYP/6-311+G(2d,p) ZPVE-corrected energy of the reactant **a-^{3}2** relative to the transition state **TS2**. The horizontal arrow designates the barrier width considering the ZPVE-corrected energy values of the stationary points superimposed with the pure electronic IRC energy profile.

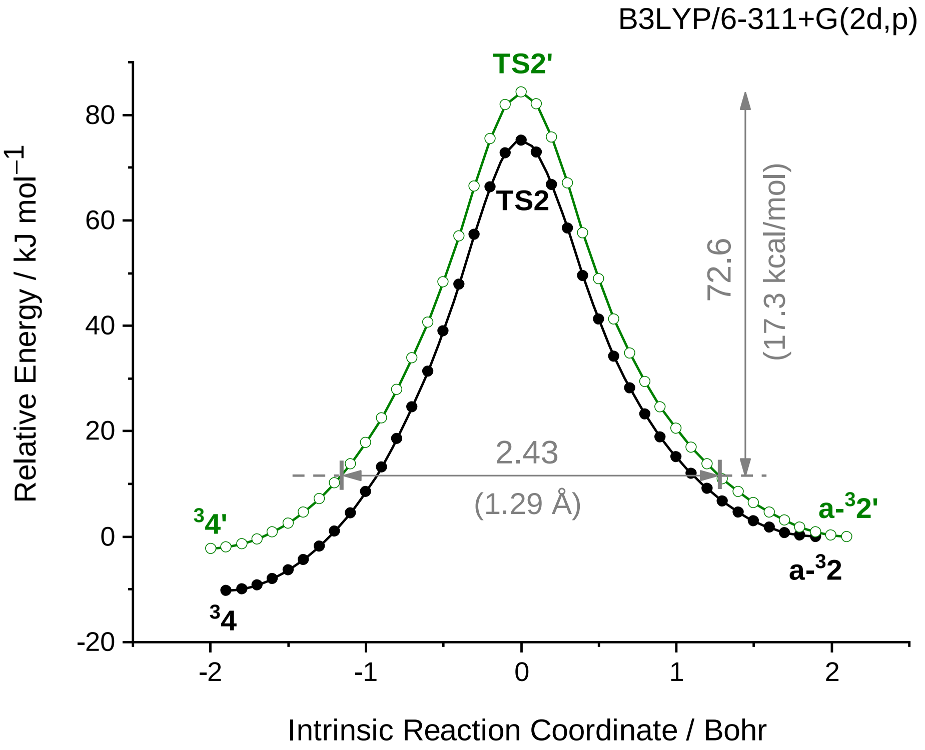

Figure S14. Relative electronic energy as a function of intrinsic reaction coordinate (IRC) for the H-shift reaction from **a-^{3}2'** to ^{3}**4'** computed at the B3LYP/6-311+G(2d,p) level in non-mass-weighted (Cartesian) coordinates (open circle, green). The vertical arrow designates the B3LYP/6-311+G(2d,p) ZPVE-corrected energy of the reactant **a-^{3}2'** relative to the transition state **TS2'**. The horizontal arrow designates the barrier width considering the ZPVE-corrected energy values of the stationary points superimposed with the pure electronic IRC energy profile. The electronic energy as a function of IRC for the H-shift reaction from **a-^{3}2** to ^{3}**4** computed at the same method is shown for comparative proposes (closed circles, black).

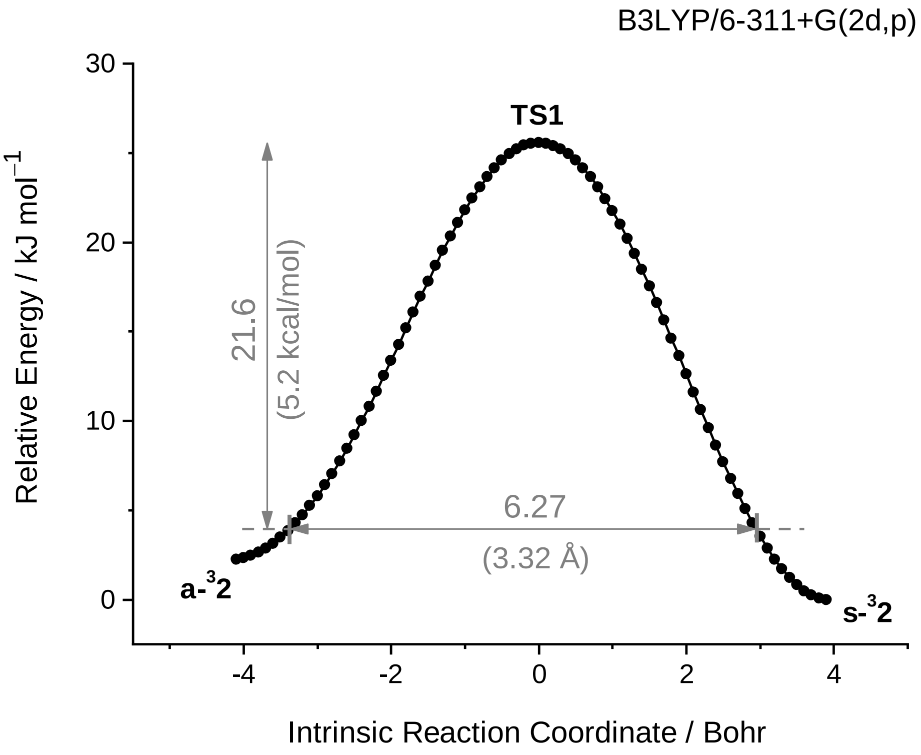

Figure S15. Relative electronic energy as a function of intrinsic reaction coordinate (IRC) for the conformation isomerization of **a-^{3}2** to **s-^{3}2** computed at the B3LYP/6-311+G(2d,p) level in non-mass-weighted (Cartesian) coordinates (closed circle). The vertical arrow designates the B3LYP/6-311+G(2d,p) ZPVE-corrected energy of the reactant **a-^{3}2** relative to the transition state **TS1**. The horizontal arrow designates the barrier width considering the ZPVE-corrected energy values of the stationary points superimposed with the pure electronic IRC energy profile.

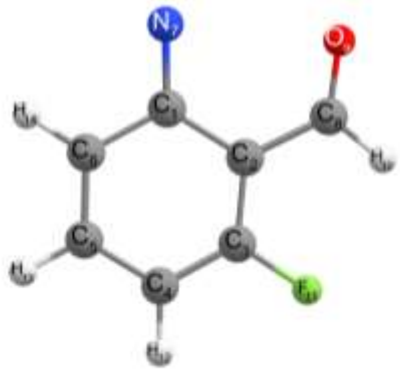

s-^{3}2

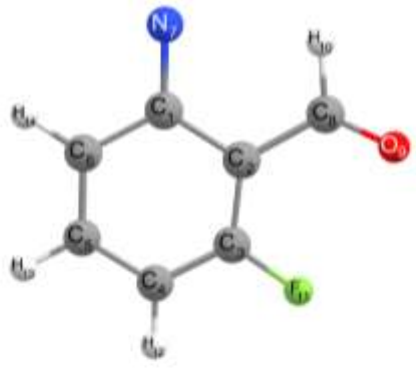

a-^{3}2

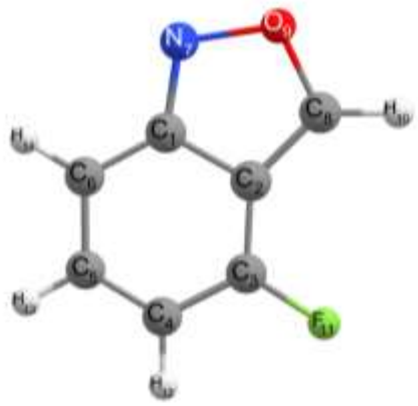

3

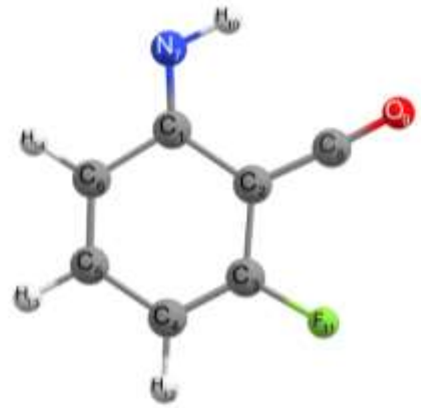

4

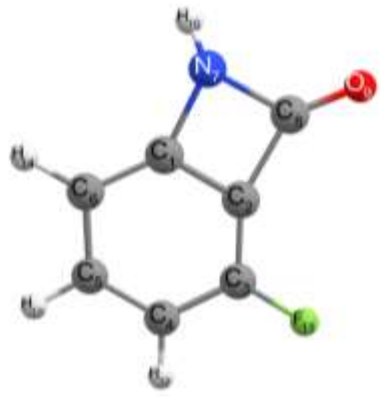

5

Figure S16. Geometry and atom numbering of species **a-^{3}2**, **3**, **4** and **5** used for the definition of internal coordinates. Color codes: red – oxygen; blue – nitrogen; grey – carbon; white – hydrogen; green – fluorine.

2. Tables

Table S1. Relative Gibbs energy at 298.15 K (ΔG_{298K} in kcal mol^{-1}) computed at the B3LYP/6-311+G(2d,p) and CBS-QB3 levels of theory for conformers of 2-formyl-3-fluorophenylazide **1** and 2-formylphenylazide **1'** and their equilibrium populations at 298.15 K (Pop_{298K} in %).[a]

Structure<br>**1', 1**<br>R = F , H	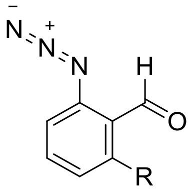	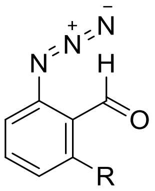	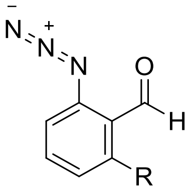	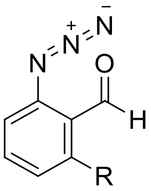
**Name**	**aa-1** | **aa-1'**	**sa-1** | **sa-1'**	**as-1** | **as-1'**	**ss-1** | **ss-1'**
ΔG_{298K} (B3LYP)	0.0 | 0.0	4.4 | 4.1	0.3 | 2.5	1.3 | 3.4
ΔG_{298K} (CBS-QB3)	0.0 | 0.0	3.5 | 3.6	0.4 | 2.7	0.6 | 2.9
Pop_{298K}	**55** | **98**	**0** | **0**	**26** | **1**	**19** | **1**

[a]Equilibrium populations were estimated from Boltzmann distribution based on the ΔG_{298K} value computed at the CBS-QB3 level of theory. In the names of structures, **a** stands for *anti* and **s** stands for *syn*, which corresponds to the orientation of the dihedral angle involving N_3 (first letter) and CHO (second letter) moieties. These computed data were published in ref. S3. Note that in the **aa-1** and **sa-1** conformers the aldehyde adopts *anti* orientation and the simplified notation mentioned in the text is **a-1**, whereas in the **as-1** and **ss-1** conformers the aldehyde adopts *syn* orientation and the simplified notation mentioned in the text is **s-1**.

Table S2. Definition of internal coordinates used in the normal mode analysis of triplet 2-formyl-3-fluorophenylnitrene **s-**^{3}**2** and **a-**^{3}**2**.[a]

Coordinate	Definition	Approximate description
S_1	$(3^{-1/2})(r_{4,12} + r_{5,13} + r_{6,14})$	ν_a(CH)
S_2	$(2^{-1/2})(r_{4,12} - r_{6,14})$	ν_b(CH)
S_3	$(6^{-1/2})(- r_{4,12} + 2r_{5,13} - r_{6,14})$	ν_c(CH)
S_4	$r_{8,9}$	ν(C8=O9)
S_5	$r_{8,10}$	ν(C8-H10)
S_6	$r_{1,7}$	ν(C1-N7)
S_7	$r_{2,8}$	ν(C2-C8)
S_8	$r_{3,11}$	ν(C3-F11)
S_9	$(12^{-1/2})(- r_{1,2} - r_{2,3} + 2r_{3,4} - r_{4,5} - r_{5,6} + 2r_{6,1})$	ν_a(CC)
S_{10}	$(4^{-1/2})(r_{1,2} - r_{2,3} + r_{4,5} - r_{5,6})$	ν_b(CC)
S_{11}	$(4^{-1/2})(r_{1,2} + r_{2,3} - r_{4,5} - r_{5,6})$	ν_c(CC)
S_{12}	$(12^{-1/2})(- r_{1,2} + r_{2,3} + 2r_{3,4} + r_{4,5} - r_{5,6} - 2r_{6,1})$	ν_d(CC)
S_{13}	$(6^{-1/2})(r_{1,2} - r_{2,3} + r_{3,4} - r_{4,5} + r_{5,6} - r_{6,1})$	ν_e(CC)
S_{14}	$(6^{-1/2})(r_{1,2} + r_{2,3} + r_{3,4} + r_{4,5} + r_{5,6} + r_{6,1})$	ν_f(CC)
S_{15}	$(2^{-1/2})(\beta_{2,10,8} - \beta_{9,10,8})$	δ(C8-H10)
S_{16}	$(2^{-1/2})(2\beta_{9,2,8} - \beta_{10,2,8} - \beta_{10,9,8})$	δ(C8=O9)
S_{17}	$(2^{-1/2})(\beta_{2,7,1} - \beta_{6,7,1})$	δ(CCN)
S_{18}	$(2^{-1/2})(\beta_{3,8,2} - \beta_{1,8,2})$	δ(CCC)
S_{19}	$(2^{-1/2})(\beta_{2,11,3} - \beta_{4,11,3})$	δ(C3-F11)
S_{20}	$(6^{-1/2})(\beta_{1,14,6} - \beta_{5,14,6} + \beta_{6,13,5} - \beta_{4,13,5} + \beta_{5,12,4} - \beta_{3,12,4})$	δ_a(CH)
S_{21}	$(4^{-1/2})(\beta_{1,14,6} - \beta_{5,14,6} - \beta_{5,12,4} + \beta_{3,12,4})$	δ_b(CH)
S_{22}	$(12^{-1/2})(-\beta_{1,14,6} + \beta_{5,14,6} + 2\beta_{6,13,5} - 2\beta_{4,13,5} - \beta_{5,12,4} + \beta_{3,12,4})$	δ_c(CH)
S_{23}	$(6^{-1/2})(\beta_{1,3,2} - \beta_{2,4,3} + \beta_{3,5,4} - \beta_{4,6,5} + \beta_{5,1,6} - \beta_{6,2,1})$	δ_a(ring)
S_{24}	$(12^{-1/2})(2\beta_{1,3,2} - \beta_{2,4,3} - \beta_{3,5,4} + 2\beta_{4,6,5} - \beta_{5,1,6} - \beta_{6,2,1})$	δ_b(ring)
S_{25}	$(4^{-1/2})(\beta_{2,4,3} - \beta_{3,5,4} + \beta_{5,1,6} - \beta_{6,2,1})$	δ_c(ring)
S_{26}	$(2^{-1/2})(\tau_{9,8,2,1} + \tau_{9,8,2,3})$	τ(C8=O9)
S_{27}	$(2^{-1/2})(\tau_{10,8,2,1} + \tau_{10,8,2,3})$	τ(C8-H10)
S_{28}	$(6^{-1/2})(\tau_{1,2,3,4} - \tau_{2,3,4,5} + \tau_{3,4,5,6} - \tau_{4,5,6,1} + \tau_{5,6,1,2} - \tau_{6,1,2,3})$	τ_a(ring)
S_{29}	$(12^{-1/2})(-\tau_{1,2,3,4} + 2\tau_{2,3,4,5} - \tau_{3,4,5,6} - \tau_{4,5,6,1} + 2\tau_{5,6,1,2} - \tau_{6,1,2,3})$	τ_b(ring)
S_{30}	$(4^{-1/2})(\tau_{1,2,3,4} - \tau_{3,4,5,6} + \tau_{4,5,6,1} - \tau_{6,1,2,3})$	τ_c(ring)
S_{31}	$\gamma_{7,6,1,2}$	γ(C1-N7)
S_{32}	$\gamma_{11,4,3,2}$	γ(C3-F11)
S_{33}	$\gamma_{8,1,2,3}$	γ(C2-C8)
S_{34}	$(3^{-1/2})(\gamma_{12,3,4,5} + \gamma_{13,4,5,6} + \gamma_{14,5,6,1})$	γ_a(CH)
S_{35}	$(6^{-1/2})(- \gamma_{12,3,4,5} + 2\gamma_{13,4,5,6} - \gamma_{14,5,6,1})$	γ_b(CH)
S_{36}	$(2^{-1/2})(\gamma_{12,3,4,5} - \gamma_{14,5,6,1})$	γ_c(CH)

[a]Abbreviations: ν = stretching, δ = in-plane bending, γ = out-of-plane bending, τ = torsion. See Figure S16 for the atom numbering scheme; $r_{i,j}$ is the distance between atoms A_i and A_j; $\beta_{i,j,k}$ is the angle between vectors A_kA_i and A_kA_j; $\beta_{i,j,k,l}$ is the angle between vector A_kA_i and A_kA_j in the plane defined by A_i, A_k and A_l atoms; $\tau_{i,j,k,l}$ is the dihedral angle between the plane defined by A_i, A_j, A_k and the plane defined by A_j, A_k and A_l atoms; $\gamma_{i,j,k,l}$ is the angle between the vector A_kA_i and the plane defined by atoms A_j, A_k, A_l. The combinations [(+), (+)] and [(+), (−)] denote in-phase and in-opposite-phase couplings between coordinates of different types.

Table S3. Definition of internal coordinates used in the normal mode analysis of 4-fluoro-2,1-benzisoxazole **3**.[a]

Coordinate	Definition	Approximate description
S_1	$r_{8,10}$	ν(C8-H10)
S_2	$(3^{-1/2})(r_{4,12} + r_{5,13} + r_{6,14})$	ν_a(CH)
S_3	$(2^{-1/2})(r_{4,12} - r_{6,14})$	ν_b(CH)
S_4	$(6^{-1/2})(- r_{4,12} + 2r_{5,13} - r_{6,14})$	ν_c(CH)
S_5	$r_{3,11}$	ν(C3-F11)
S_6	$(2^{-1/2})(r_{1,2} + r_{4,5})$	ν_a(CC)
S_7	$(2^{-1/2})(r_{1,2} - r_{4,5})$	ν_b(CC)
S_8	$(2^{-1/2})(r_{2,3} + r_{5,6})$	ν_c(CC)
S_9	$(2^{-1/2})(r_{2,3} - r_{5,6})$	ν_d(CC)
S_{10}	$(2^{-1/2})(r_{3,4} + r_{6,1})$	ν_e(CC)
S_{11}	$(2^{-1/2})(r_{3,4} - r_{6,1})$	ν_f(CC)
S_{12}	$r_{1,7}$	ν(C1-N7)
S_{13}	$r_{2,8}$	ν(C2-C8)
S_{14}	$r_{7,9}$	ν(N7-O9)
S_{15}	$r_{8,9}$	ν(C8-O9)
S_{16}	$(6^{-1/2})(\beta_{5,1,6} - \beta_{6,2,1} + \beta_{1,3,2} - \beta_{2,4,3} + \beta_{3,5,4} - \beta_{4,6,5})$	δ_a(Bz-ring)
S_{17}	$(12^{-1/2})(2\beta_{5,1,6} - \beta_{6,2,1} - \beta_{1,3,2} + 2\beta_{2,4,3} - \beta_{3,5,4} - \beta_{4,6,5})$	δ_b(Bz-ring)
S_{18}	$(4^{-1/2})(\beta_{6,2,1} - \beta_{1,3,2} + \beta_{3,5,4} - \beta_{4,6,5})$	δ_c(Bz-ring)
S_{19}	$((1+2a^2+2b^2)^{-1/2})(\beta_{7,8,9} + a(\beta_{1,9,7} + \beta_{9,2,8}) + b(\beta_{2,7,1} + \beta_{8,1,2}))$	δ_a(Is-ring)
S_{20}	$(2(a-b)^2 + 2(1-a)^2)^{-1/2})((a-b)(\beta_{1,9,7} - \beta_{9,2,8}) + (1-a)(\beta_{2,7,1} - \beta_{8,1,2}))$	δ_b(Is-ring)
S_{21}	$(2^{-1/2})(\beta_{9,10,8} - \beta_{2,10,8})$	δ(C8-H10)
S_{22}	$(2^{-1/2})(\beta_{2,11,3} - \beta_{4,11,3})$	δ(C3-F11)
S_{23}	$(6^{-1/2})(\beta_{1,14,6} - \beta_{5,14,6} + \beta_{6,13,5} - \beta_{4,13,5} + \beta_{5,12,4} - \beta_{3,12,4})$	δ_a(CH)
S_{24}	$(4^{-1/2})(\beta_{1,14,6} - \beta_{5,14,6} - \beta_{5,12,4} + \beta_{3,12,4})$	δ_b(CH)
S_{25}	$(12^{-1/2})(- \beta_{1,14,6} + \beta_{5,14,6} + 2\beta_{6,13,5} - 2\beta_{4,13,5} - \beta_{5,12,4} + \beta_{3,12,4})$	δ_c(CH)
S_{26}	$(6^{-1/2})(\tau_{6,5,4,3} - \tau_{5,4,3,2} + \tau_{4,3,2,1} - \tau_{3,2,1,6} + \tau_{2,1,6,5} - \tau_{1,6,5,4})$	τ_a(Bz-ring)
S_{27}	$(12^{-1/2})(2\tau_{6,5,4,3} - \tau_{5,4,3,2} - \tau_{4,3,2,1} + 2\tau_{3,2,1,6} - \tau_{2,1,6,5} - \tau_{1,6,5,4})$	τ_b(Bz-ring)
S_{28}	$(4^{-1/2})(\tau_{5,4,3,2} - \tau_{4,3,2,1} + \tau_{2,1,6,5} - \tau_{1,6,5,4})$	τ_c(Bz-ring)
S_{29}	$((1+2a^2 + 2b^2)^{-1/2})(\tau_{7,1,2,8} + b(\tau_{8,9,7,1} + \tau_{2,8,9,7}) + a(\tau_{9,7,1,2} + \tau_{1,2,8,9}))$	τ_a(Is-ring)
S_{30}	$(2(a-b)^2 + 2(1-a)^2)^{-1/2})((a-b)(\tau_{1,2,8,9} - \tau_{9,7,1,2}) + (1-a)(\tau_{2,8,9,7} - \tau_{8,9,7,1}))$	τ_b(Is-ring)
S_{31}	$(2^{-1/2})(\tau_{8,2,1,6} - \tau_{3,2,1,7})$	τ(Bz-Ox)
S_{32}	$\gamma_{10,2,8,9}$	γ(C8-H10)
S_{33}	$\gamma_{11,4,3,2}$	γ(C3-F11)
S_{34}	$(3^{-1/2})(\gamma_{12,3,4,5} + \gamma_{13,4,5,6} + \gamma_{14,5,6,1})$	γ_a(CH)
S_{35}	$(6^{-1/2})(- \gamma_{12,3,4,5} + 2\gamma_{13,4,5,6} - \gamma_{14,5,6,1})$	γ_b(CH
S_{36}	$(2^{-1/2})(\gamma_{12,3,4,5} - \gamma_{14,5,6,1})$	γ_c(CH)

[a]Abbreviations: ν = stretching, δ = in-plane bending, γ = out-of-plane bending, τ = torsion, Bz = benzene ring, Is = isoxazole ring. See Figure S16 for the atom numbering scheme; $r_{i,j}$ is the distance between atoms A_i and A_j; $\beta_{i,j,k}$ is the angle between vectors A_kA_i and A_kA_j; $\beta_{i,j,k,l}$ is the angle between vector A_kA_i and A_kA_j in the plane defined by A_i, A_k and A_l atoms; $\tau_{i,j,k,l}$ is the dihedral angle between the plane defined by A_i, A_j, A_k and the plane defined by A_j, A_k and A_l atoms; $\gamma_{i,j,k,l}$ is the angle between the vector A_kA_i and the plane defined by atoms A_j, A_k, A_l. The combinations [(+), (+)] and [(+), (−)] denote in-phase and in-opposite-phase couplings between coordinates of different types.

Table S4. Definition of internal coordinates used in the normal mode analysis of 2-fluoro-6-imino-2,4-cyclohexadien-1-ketene **4**.[a]

Coordinate	Definition	Approximate description
S_1	$r_{7,10}$	ν(NH)
S_2	$(3^{-1/2})(r_{4,12} + r_{5,13} + r_{6,14})$	ν_a(CH)
S_3	$(2^{-1/2})(r_{4,12} - r_{6,14})$	ν_b(CH)
S_4	$(6^{-1/2})(- r_{4,12} + 2r_{5,13} - r_{6,14})$	ν_c(CH)
S_5	$(2^{-1/2})(r_{2,8} - r_{8,9})$	ν(C=C=O)as
S_6	$(2^{-1/2})(r_{2,8} + r_{8,9})$	ν(C=C=O)s
S_7	$r_{1,7}$	ν(C=N)
S_8	$(2^{-1/2})(r_{3,4} - r_{5,6})$	ν(C=C)as
S_9	$(2^{-1/2})(r_{3,4} + r_{5,6})$	ν(C=C)s
S_{10}	$(2^{-1/2})(r_{6,1} + r_{1,2})$	ν_a(C–C)s
S_{11}	$(2^{-1/2})(r_{6,1} - r_{1,2})$	ν_a(C–C)as
S_{12}	$(2^{-1/2})(r_{2,3} + r_{4,5})$	ν_b(C–C)s
S_{13}	$(2^{-1/2})(r_{2,3} - r_{4,5})$	ν_b(C–C)as
S_{14}	$r_{3,11}$	ν(C3-F11)
S_{15}	$(6^{-1/2})(\beta_{1,14,6} - \beta_{5,14,6} + \beta_{6,13,5} - \beta_{4,13,5} + \beta_{5,12,4} - \beta_{3,12,4})$	δ_a(CH)
S_{16}	$(4^{-1/2})(\beta_{1,14,6} - \beta_{5,14,6} - \beta_{5,12,4} + \beta_{3,12,4})$	δ_b(CH)
S_{17}	$(12^{-1/2})(- \beta_{1,14,6} + \beta_{5,14,6} + 2\beta_{6,13,5} - 2\beta_{4,13,5} - \beta_{5,12,4} + \beta_{3,12,4})$	δ_c(CH)
S_{18}	$\beta_{1,10,7}$	δ(CNH)
S_{19}	$(2^{-1/2})(\beta_{2,11,3} - \beta_{4,11,3})$	δ(C3-F11)
S_{20}	$(2^{-1/2})(\beta_{2,7,1} - \beta_{6,7,1})$	δ(CCN)
S_{21}	$(2^{-1/2})(\beta_{9,1,8} - \beta_{9,3,8})$	δ(CCO)
S_{22}	$(2^{-1/2})(\beta_{8,1,2} - \beta_{8,3,2})$	δ(CCC)
S_{23}	$(6^{-1/2})(\beta_{2,6,1} - \beta_{1,5,6} + \beta_{6,4,5} - \beta_{5,3,4} + \beta_{4,2,3} - \beta_{3,1,2})$	δ_a(ring)
S_{24}	$(4^{-1/2})(\beta_{2,6,1} - \beta_{1,5,6} + \beta_{5,3,4} - \beta_{4,2,3})$	δ_b(ring)
S_{25}	$(12^{-1/2})(- \beta_{2,6,1} - \beta_{1,5,6} + 2\beta_{6,4,5} - \beta_{5,3,4} - \beta_{4,2,3} + 2\beta_{3,1,2})$	δ_c(ring)
S_{26}	$\beta_{9,2,8}$	δ(CCN)
S_{27}	$(2^{-1/2})(\tau_{10,7,1,2} + \tau_{10,7,1,6})$	τ(NH)
S_{28}	$(6^{-1/2})(\tau_{1,2,3,4} - \tau_{2,3,4,5} + \tau_{3,4,5,6} - \tau_{4,5,6,1} + \tau_{5,6,1,2} - \tau_{6,1,2,3})$	τ_a(ring)
S_{29}	$(12^{-1/2})(- \tau_{1,2,3,4} + 2\tau_{2,3,4,5} - \tau_{3,4,5,6} - \tau_{4,5,6,1} + 2\tau_{5,6,1,2} - \tau_{6,1,2,3})$	τ_b(ring)
S_{30}	$(4^{-1/2})(\tau_{1,2,3,4} - \tau_{3,4,5,6} + \tau_{4,5,6,1} - \tau_{6,1,2,3})$	τ_c(ring)
S_{31}	$\gamma_{7,6,1,2}$	γ(C=N)
S_{32}	$\gamma_{8,1,2,3}$	γ(CC)
S_{33}	$\gamma_{11,2,3,4}$	γ(CF)
S_{34}	$(3^{-1/2})(\gamma_{12,3,4,5} + \gamma_{13,4,5,6} + \gamma_{14,5,6,1})$	γ_a(CH)
S_{35}	$(6^{-1/2})(- \gamma_{12,3,4,5} + 2\gamma_{13,4,5,6} - \gamma_{14,5,6,1})$	γ_b(CH)
S_{36}	$(2^{-1/2})(\gamma_{12,3,4,5} - \gamma_{14,5,6,1})$	γ_c(CH)

[a] Abbreviations: ν = stretching, δ = in-plane bending, γ = out-of-plane bending, τ = torsion. See Figure S16 for the atom numbering scheme; $r_{i,j}$ is the distance between atoms A_i and A_j; $\beta_{i,j,k}$ is the angle between vectors A_kA_i and A_kA_j; $\beta_{i,j,k,l}$ is the angle between vector A_kA_i and A_kA_j in the plane defined by A_i, A_k and A_l atoms; $\tau_{i,j,k,l}$ is the dihedral angle between the plane defined by A_i, A_j, A_k and the plane defined by A_j, A_k and A_l atoms; $\gamma_{i,j,k,l}$ is the angle between the vector A_kA_i and the plane defined by atoms A_j, A_k, A_l. The combinations [(+), (+)] and [(+), (−)] denote in-phase and in-opposite-phase couplings between coordinates of different types.

Table S5. Definition of internal coordinates used in the normal mode analysis of 3-fluorobenzoazetinone **5**.[a]

Coordinate	Definition	Approximate description
S_1	$r_{7,10}$	ν(N7-H10)
S_2	$(3^{-1/2})(r_{4,12} + r_{5,13} + r_{6,14})$	ν_a(CH)
S_3	$(2^{-1/2})(r_{4,12} - r_{6,14})$	ν_b(CH)
S_4	$(6^{-1/2})(- r_{4,12} + 2r_{5,13} - r_{6,14})$	ν_c(CH)
S_5	$r_{8,9}$	ν(C8=O9)
S_6	$r_{3,11}$	ν(C3-F11)
S_7	$(12^{-1/2})(- r_{5,6} - r_{6,1} + 2r_{1,2} - r_{2,3} - r_{3,4} + 2r_{4,5})$	ν_a(CC)
S_8	$(4^{-1/2})(r_{5,6} - r_{6,1} + r_{2,3} - r_{3,4})$	ν_b(CC)
S_9	$(4^{-1/2})(r_{5,6} + r_{6,1} - r_{2,3} - r_{3,4})$	ν_c(CC)
S_{10}	$(12^{-1/2})(- r_{5,6} + r_{6,1} + 2r_{1,2} + r_{2,3} - r_{3,4} - 2r_{4,5})$	ν_d(CC)
S_{11}	$(6^{-1/2})(r_{5,6} - r_{6,1} + r_{1,2} - r_{2,3} + r_{3,4} - r_{4,5})$	ν_e(CC)
S_{12}	$(6^{-1/2})(r_{5,6} + r_{6,1} + r_{1,2} + r_{2,3} + r_{3,4} + r_{4,5})$	ν_f(CC)
S_{13}	$r_{1,7}$	ν(C1-N7)
S_{14}	$r_{2,8}$	ν(C2-C8)
S_{15}	$r_{7,8}$	ν(N7-C8)
S_{16}	$(6^{-1/2})(\beta_{5,1,6} - \beta_{6,2,1} + \beta_{1,3,2} - \beta_{2,4,3} + \beta_{3,5,4} - \beta_{4,6,5})$	δ_a(Bz-ring)
S_{17}	$(12^{-1/2})(2\beta_{5,1,6} - \beta_{6,2,1} - \beta_{1,3,2} + 2\beta_{2,4,3} - \beta_{3,5,4} - \beta_{4,6,5})$	δ_b(Bz-ring)
S_{18}	$(4^{-1/2})(\beta_{6,2,1} - \beta_{1,3,2} + \beta_{3,5,4} - \beta_{4,6,5})$	δ_c(Bz-ring)
S_{19}	$(4^{-1/2})(\beta_{2,7,1} - \beta_{1,8,7} + \beta_{7,2,8} - \beta_{8,1,2})$	δ(Az-ring)
S_{20}	$(2^{-1/2})(\beta_{9,7,8} - \beta_{9,2,8})$	δ(C8=O9)
S_{21}	$\beta_{10,1,7}$	δ(N7-H10)
S_{22}	$(2^{-1/2})(\beta_{2,11,3} - \beta_{4,11,3})$	δ(C3-F11)
S_{23}	$(6^{-1/2})(\beta_{1,14,6} - \beta_{5,14,6} + \beta_{6,13,5} - \beta_{4,13,5} + \beta_{5,12,4} - \beta_{3,12,4})$	δ_a(CH)
S_{24}	$(4^{-1/2})(\beta_{1,14,6} - \beta_{5,14,6} - \beta_{5,12,4} + \beta_{3,12,4})$	δ_b(CH)
S_{25}	$(12^{-1/2})(- \beta_{1,14,6} + \beta_{5,14,6} + 2\beta_{6,13,5} - 2\beta_{4,13,5} - \beta_{5,12,4} + \beta_{3,12,4})$	δ_c(CH)
S_{26}	$(6^{-1/2})(\tau_{6,5,4,3} - \tau_{5,4,3,2} + \tau_{4,3,2,1} - \tau_{3,2,1,6} + \tau_{2,1,6,5} - \tau_{1,6,5,4})$	τ_a(Bz-ring)
S_{27}	$(12^{-1/2})(2\tau_{6,5,4,3} - \tau_{5,4,3,2} - \tau_{4,3,2,1} + 2\tau_{3,2,1,6} - \tau_{2,1,6,5} - \tau_{1,6,5,4})$	τ_b(Bz-ring)
S_{28}	$(4^{-1/2})(\tau_{5,4,3,2} - \tau_{4,3,2,1} + \tau_{2,1,6,5} - \tau_{1,6,5,4})$	τ_c(Bz-ring)
S_{29}	$(4^{-1/2})(\tau_{1,7,8,2} - \tau_{7,8,2,1} + \tau_{8,2,1,7} - \tau_{2,1,7,8})$	τ(Az-ring)
S_{30}	$(2^{-1/2})(\tau_{8,2,1,6} - \tau_{3,2,1,7})$	τ(Bz-Az)
S_{31}	$\gamma_{9,7,8,2}$	γ(C8=O9)
S_{32}	$\gamma_{10,1,7,8}$	γ(N7-H10)
S_{33}	$\gamma_{11,4,3,2}$	γ(C3-F11)
S_{34}	$(3^{-1/2})(\gamma_{12,3,4,5} + \gamma_{13,4,5,6} + \gamma_{14,5,6,1})$	γ_a(CH)
S_{35}	$(6^{-1/2})(- \gamma_{12,3,4,5} + 2\gamma_{13,4,5,6} - \gamma_{14,5,6,1})$	γ_b(CH)
S_{36}	$(2^{-1/2})(\gamma_{12,3,4,5} - \gamma_{14,5,6,1})$	γ_c(CH)

[a] Abbreviations: ν = stretching, δ = in-plane bending, γ = out-of-plane bending, τ = torsion, Bz = benzene ring, Az = 2-azetidinone ring. See Figure S16 for the atom numbering scheme; $r_{i,j}$ is the distance between atoms A_i and A_j; $\beta_{i,j,k}$ is the angle between vectors A_kA_i and A_kA_j; $\beta_{i,j,k,l}$ is the angle between vector A_kA_i and A_kA_j in the plane defined by A_i, A_k and A_l atoms; $\tau_{i,j,k,l}$ is the dihedral angle between the plane defined by A_i, A_j, A_k and the plane defined by A_j, A_k and A_l atoms; $\gamma_{i,j,k,l}$ is the angle between the vector A_kA_i and the plane defined by atoms A_j, A_k, A_l. The combinations [(+), (+)] and [(+), (−)] denote in-phase and in-opposite-phase couplings between coordinates of different types.

Table S6. Experimental IR spectral data (nitrogen matrix at 10 K), B3LYP/6-311+G(2d,p) computed vibrational frequencies ($\tilde{\nu}$, cm^{-1}), absolute infrared intensities (A^{th}, km mol^{-1}), and vibrational assignment (PED, %) of triplet 2-formyl-3-fluorophenylnitrene **s-^{3}2**.

Experimental[a]			Calculated[b]		PED[c]
$\tilde{\nu}$ N_2/10 K	$\tilde{\nu}$ Ar/15 K[S1]	I	$\tilde{\nu}$	A^{th}	
2886	-	w	2899	58.5	100 [ν(C8-H10)]
1697	1695	s	1705	191.7	90 [ν(C8=O9)]
1529	1528/1526	m/m	1547	23.9	34 [ν_b(CC)] ; 29 [ν_a(CC)]
1519	1517	w(ov)	1519	83.6	25 [δ_b(CH)] ; 22 [ν_b(CC)] ; 19 [ν_a(CC)] ; 12 [ν_c(CC)]
-	1409	vw	1428	8.4	37 [δ_a(CH)] ; 29 [ν_e(CC)] ; 19 [δ(C8-H10)]
1396	1393	s	1394	44.4	55 [δ(C8-H10)] ; 18 [δ_a(CH)] ; 10 [ν_d(CC)]
1384	1383	m	1374	34.9	26 [ν_c(CC)] ; 17 [δ_b(CH)] ; 12 [ν_a(CC)] ; 11 [δ(C8-H10)]
1294	1296	w	1288	26.9	59 [ν(C1-N7)] ; 16 [δ_a(ring)] ; 12 [ν_e(CC)]
1289	1288	m	1283	46.4	89 [ν_e(CC)] ; 13 [δ_a(CH)]
1227/1222	1226/1220	m/m	1206	46.1	69 [ν_d(CC)] ; 28 [ν(C3-F11)] ; 20 [δ_a(CH)]
1164					
1149	1147	w	1151	6.4	57 [δ_c(CH)] ; 17 [ν_b(CC)]
1134	1138/1132	m	1127	73.6	22 [δ_b(CH)] ; 21 [ν(C2-C8)] ; 17 [ν_a(CC)] ; 13 [ν(C1-N7)]
-	-	-	1051	3.8	28 [ν_c(CC)] ; 28 [ν_f(CC)] ; 20 [δ_b(CH)]
1036	1035	m	1027	36.5	35 [ν_d(CC)] ; 25 [ν(C3-F11)]
-	-	-	991	0.0	70 [τ(C8-H10)] ; 23 [τ(C8=O9)]
-	-	-	961	0.0	118 [γ_b(CH)]
-	-	-	866	0.1	101 [γ_c(CH)]
821	822/820	w/m	813	23.3	43 [δ_a(ring)] ; 15 [δ_b(ring)] ; 14 [ν(C2-C8)]
781/779	781/776	w/s	778	46.5	64 [γ_a(CH)] ; 15 [γ(C3-F11)] ; 13 [γ(C1-N7)]
705	704	w	698	10.9	36 [ν_f(CC)] ; 21 [δ(C8=O9)]
681	679	vw	682	15.8	50 [τ_a(ring)] ; 34 [γ_a(CH)] ; 11 [γ(C1-N7)]

[a]Experimental intensities (I) are given in qualitative terms: s = strong; m = medium; w = weak; vw = very weak; ov = overlapped. The spectral range in 1700−660 cm^{-1} region plus the aldehyde ν(C-H) band is reported. [b]Computed harmonic wavenumbers were multiplied by 0.979 scale factor and are expressed in cm^{-1}. [c]Abbreviations: ν = stretching, δ = in-plane bending, γ = out-of-plane bending, τ = torsion. PED values lower than 10% are not included. Definition of internal coordinates is given in Table S2.

Table S7. Experimental IR spectral data (nitrogen matrix at 10 K), B3LYP/6-311+G(2d,p) computed vibrational frequencies ($\tilde{\nu}$, cm^{-1}), absolute infrared intensities (A^{th}, km mol^{-1}), and vibrational assignment (PED, %) of triplet 2-formyl-3-fluorophenylnitrene **a-^{3}2**.

Experimental[a]		Calculated[b]		PED[c]
$\tilde{\nu}$	I	$\tilde{\nu}$	A^{th}	
2851	w	2858	43.2	100 [ν(C8-H10)]
1713	s	1725	285.6	88 [ν(C8=O9)]
1542	w	1540	50.6	30 [ν_a(CC)] ; 29 [ν_b(CC)]
1532	m	1522	40.7	26 [ν_b(CC)] ; 21 [δ_b(CH)] ; 15 [ν_a(CC)] ; 12 [ν_c(CC)]
1426	w	1426	20.2	39 [δ_a(CH)] ; 17 [ν_d(CC)] ; 14 [δ(C8-H10)]
1397	vw	1396	19.4	61 [δ(C8-H10)]
1363	vw	1363	26.5	18 [ν_c(CC)] ; 13 [ν_e(CC)] ; 13 [δ_b(CH)] ; 13 [δ_a(CH)] ; 10 [δ(C8-H10)]
1302	m	1295	28.3	53 [ν(C1-N7)] ; 17 [δ_a(ring)]
1280	m	1268	31.5	40 [ν_e(CC)] ; 13 [ν(C3-F11)] ; 13 [δ_a(CH)]
1233	vw	1216	30.4	27 [ν(C3-F11)] ; 22 [ν_e(CC)] ; 15 [δ_a(CH)] ; 11 [ν_d(CC)]
1174	w	1165	34.3	42 [δ_c(CH)] ; 12 [ν_e(CC)] ; 10 [ν_b(CC)]
1123	m	1115	52.1	19 [δ_c(CH)] ; 17 [ν(C1-N7)] ; 15 [ν(C2-C8)] ; 15 [δ_b(CH)] ; 10 [ν_c(CC)]
1065	vw	1059	10.3	27 [ν_c(CC)] ; 23 [δ_b(CH)] ; 12 [ν_f(CC)]
1024	m	1017	29.0	28 [ν_d(CC)] ; 22 [ν_f(CC)] ; 14 [ν(C3-F11)]
-	-	1000	0.0	69 [τ(C8-H10)] ; 24 [τ(C8=O9)]
-	-	962	0.0	119 [γ_b(CH)]
-	-	867	0.6	100 [γ_c(CH)]
828	m	821	25.0	44 [δ_a(ring)] ; 18 [ν(C2-C8)] ; 12 [δ_b(ring)]
782	s	780	46.0	62 [γ_a(CH)] ; 15 [γ(C3-F11)] ; 14 [γ(C1-N7)]
727 ?		717	20.4	26 [ν_f(CC)] ; 19 [δ(C8=O9)] ; 12 [ν(C3-F11)]
683	w	683	16.5	49 [τ_a(ring)] ; 35 [γ_a(CH)]

[a]Experimental intensities (I) are given in qualitative terms: s = strong; m = medium; w = weak; vw = very weak. The spectral range in 1700−660 cm^{-1} region plus the aldehyde ν(C-H) band is reported. [b]Computed harmonic wavenumbers were multiplied by 0.979 scale factor and are expressed in cm^{-1}. [c]Abbreviations: ν = stretching, δ = in-plane bending, γ = out-of-plane bending, τ = torsion. PED values lower than 10% are not included. Definition of internal coordinates is given in Table S2.

Table S8. Experimental IR spectral data (nitrogen matrix at 10 K), B3LYP/6-311+G(2d,p) computed vibrational frequencies ($\tilde{\nu}$, cm^{-1}), absolute infrared intensities (A^{th}, km mol^{-1}), and vibrational assignment (PED, %) of 4-fluoro-2,1-benzisoxazole **3.**

Experimental[a]			Calculated[b]		PED[c]
$\tilde{\nu}$ N_2/10 K	$\tilde{\nu}$ Ar /15 K[S1]	I	$\tilde{\nu}$	A^{th}	
1667/1662	1666	m	1657	62.4	32 [ν_c(CC)] ; 23 [ν_e(CC)] ; 15 [ν(C2-C8)]
1582	1585/1582	w/s	1572	81.3	22 [ν(C2-C8)] ; 20 [ν_e(CC)] ; 11 [ν_f(CC)]
1521	1521	vw(ov)	1520	32.7	17 [ν_a(CC)] ; 15 [ν_c(CC)] ; 13 [ν_d(CC)] ; 13 [ν(C1-N7)] ; 13 [δ_c(CH)]
1463	1463	w	1454	15.6	33 [ν_f(CC)] ; 30 [ν_d(CC)] ; 12 [δ_b(CH)] ; 11 [δ_a(CH)]
-	-	-	1388	9.9	29 [ν(C1-N7)] ; 20 [ν_b(CC)]
1379	1380/1376	w/s	1371	65.2	18 [ν(C2-C8)] ; 18 [δ_a(CH)] ; 13 [δ(C8-H10)] ; 11 [ν_d(CC)]
-	-	-	1353	0.5	21 [ν(C1-N7)] ; 21 [δ_b(CH)] ; 19 [ν_b(CC)] ; 13 [δ_a(CH)]
-	-	-	1260	3.4	40 [δ(C8-H10)] ; 16 [ν(C8-O9)] ; 12 [ν(C2-C8)]
1250/1246	1248/1246	m/m	1238	55.9	34 [δ_a(CH)] ; 26 [ν(C3-F11)]
-	-	-	1159	0.4	65 [δ_c(CH)]
1123	1117/1115	m/w	1119	33.4	70 [ν(C8-O9)] ; 19 [δ(C8-H10)]
1076	1076	m	1067	48.6	20 [ν(C3-F11)] ; 20 [δ_b(Is-ring)] ; 10 [ν_a(CC)]
1015	-	vw	1020	10.1	33 [ν_b(CC)] ; 24 [δ_b(CH)] ; 15 [ν_a(CC)]
-	-	-	961	0.2	114 [γ_a(CH)]
926	926/925	w/w	921	7.0	64 [δ_b(Is-ring)] ; 12 [ν_b(CC)] ; 10 [ν_a(CC)]
882	880	m	878	30.7	87 [ν(N7-O9)]
-	-	-	867	2.6	92 [γ_c(CH)]
860	862/861	w	856	8.7	52 [δ_a(Bz-ring)]
-	-	-	815	3.6	70 [γ(C8-H10)] ; 12 [τ_a(Is-ring)] ; 11 [τ_a(Bz-ring)]
790	786	s	786	76.5	50 [γ_a(CH)] ; 22 [γ(C8-H10)] ; 10 [τ_a(Is-ring)]
724 ?	721	vw	721	9.9	41 [γ_a(CH)] ; 35 [τ_a(Bz-ring)] ; 18 [τ_a(Is-ring)]
-	668	m	663	15.2	30 [ν_a(CC)] ; 13[ν(C3-F11)]

[a]Experimental intensities (I) are given in qualitative terms: s = strong; m = medium; w = weak; vw = very weak; ov = overlapped. The spectral range in 1700−660 cm^{-1} region is reported. [b]Computed harmonic wavenumbers were multiplied by 0.979 scale factor and are expressed in cm^{-1}. [c]Abbreviations: ν = stretching, δ = in-plane bending, γ = out-of-plane bending, τ = torsion. PED values lower than 10% are not included. Definition of internal coordinates is given in Table S3.

Table S9. Experimental IR spectral data (nitrogen matrix at 10 K), B3LYP/6-311+G(2d,p) computed vibrational frequencies ($\tilde{\nu}$, cm^{-1}), absolute infrared intensities (A^{th}, km mol^{-1}), and vibrational assignment (PED, %) of 2-fluoro-6-imino-2,4-cyclohexadien-1-ketene **4**.

Experimental[a]		Calculated[b]		PED[c]
$\tilde{\nu}$	I	$\tilde{\nu}$	A^{th}	
2137	s	2147	1085.2	95 [ν(C=C=O)as]
1672/1663	m	1665	143.9	62 [ν(C=C)as]
1582 ?		1567	86.6	66 [ν(C=N)]
1559	s	1549	296.3	62 [ν(C=C)s]
1419	vw	1411	17.9	32 [δ_b(CH)] ; 28 [ν_b(C–C)as]
-		1387	14.9	30 [δ_a(CH)] ; 12 [ν(C=C=O)s]
1325/1322	m	1291	97.8	27 [δ_a(CH)] ; 26 [δ(CNH)] ; 17 [ν_a(C–C)as]
1271	vw	1263	10.4	41 [ν(C=C=O)s] ; 25 [δ(CNH)] ; 11 [δ_a(ring)]
1247	w	1229	18.7	27 [ν(C3-F11)] ; 18 [ν_b(C–C)s] ; 18 [δ_b(CH)] ; 14 [δ_a(CH)]
1160	m	1159	74.1	63 [δ_c(CH)]
1117	w	1104	49.8	30 [ν_a(C–C)as] ; 20 [δ(CNH)] ; 12 [ν(C3-F11)]
1008	w	1005	23.6	25 [ν_b(C–C)s] ; 23 [ν_b(C–C)as] ; 19 [δ_b(CH)]
995	vw	979	12.7	27 [ν_a(C–C)s] ; 20 [ν(C3-F11)] ; 10 [δ(CNH)]
-		969	0.0	105 [γ_b(CH)] ; 15 [γ_c(CH)]
849	vw	842	5.6	78 [γ_c(CH)] ; 13 [γ_b(CH)]
-		804	0.3	57 [τ(NH)] ; 31 [γ_a(CH)]
792	w	791	15.0	51 [δ_a(ring)] ; 14 [δ_c(ring)]
778	s	767	94.7	35 [δ_a(ring)] ; 30 [γ(C=N)] ; 26 [τ(NH)]
679	w	677	27.5	54 [τ_a(ring)] ; 28 [δ_a(ring)] ; 18 [γ(C=N)]
661	vw	652	10.7	24 [ν_b(C–C)s] ; 11 [δ(CCO)] ; 10 [ν(C=C)s] ; 10 [ν(C=C=O)s]

[a]Experimental intensities (I) are given in qualitative terms: s = strong; m = medium; w = weak; vw = very weak. The spectral range in 1700−650 cm^{-1} region plus the ν(C=C=O)$_{as}$ band is reported. [b]Computed harmonic wavenumbers were multiplied by 0.979 scale factor and are expressed in cm^{-1}. [c]Abbreviations: ν = stretching, δ = in-plane bending, γ = out-of-plane bending, τ = torsion. PED values lower than 10% are not included. Definition of internal coordinates is given in Table S4.

Table S10. Experimental IR spectral data (nitrogen matrix at 10 K), B3LYP/6-311+G(2d,p) computed vibrational frequencies ($\tilde{\nu}$, cm^{-1}), absolute infrared intensities (A^{th}, km mol^{-1}), and vibrational assignment (PED, %) of 3-fluorobenzoazetinone **5**.

Experimental[a]		Calculated[b]		PED[c]
$\tilde{\nu}$	I	$\tilde{\nu}$	A^{th}	
3418	w	3411	32.1	101 [ν(N7-H10)]
1874/1830	s	1849	567.2	86 [ν(C8=O9)]
1645	m	1632	131.0	60 [ν_a(CC)]
1627/1619	s	1615	294.1	55 [ν_b(CC)] ; 10 [ν_a(CC)]
1476	m	1473	63.6	34 [ν_c(CC)] ; 25 [δ_a(CH)] ; 15 [δ_b(CH)]
1420	vw	1422	11.6	38 [ν_d(CC)] ; 25 [δ_b(CH)] ; 20 [δ_a(CH)]
1375	vw	1368	21.8	54 [ν_e(CC)] ; 14 [ν(C1-N7)] ; 12 [δ_c(CH)]
1258	m	1249	24.2	37 [δ(N7-H10)] ; 22 [δ_a(CH)]
1258	m	1244	109.3	41 [ν(C3-F11)] ; 17 [δ_a(Bz-ring)] ; 10 [ν_f(CC)]
1219	vw	1212	14.9	43 [δ(N7-H10)] ; 21 [δ_a(CH)] ; 10 [ν_c(CC)]
-		1153	2.9	55 [δ_c(CH)] ; 21 [ν_e(CC)] ; 10 [ν_a(CC)]
-		1088	19.0	17 [δ_b(CH)] ; 11 [δ_a(Bz-ring)] ;10 [ν(C2-C8)] ; 10 [ν_c(CC)]
1096	w	1077	53.6	15 [ν_d(CC)] ; 13 [δ(Az-ring)]
-		1020	1.0	36 [ν_f(CC)] ; 16 [δ_b(CH)] ; 15 [ν_d(CC)] ; 13 [δ_a(Bz-ring)]
-		954	0.4	119 [γ_b(CH)]
931	vw	906	17.5	25 [δ_a(Bz-ring)] ; 19 [δ(C8=O9)] ; 17 [ν(N7-C8)]
		854	0.5	101 [γ_c(CH)]
798	w	806	47.7	32 [τ(Az-ring)] ; 22 [γ(C8=O9)] ; 20 [τ_a(Bz-ring)] ; 11 [γ_a(CH)] ; 10 [γ(C3-F11)]
		769	25.2	21 [ν(N7-C8)] ; 12 [γ_a(CH)] ; 10 [δ_a(Bz-ring)]
764	w	755	40.5	65 [γ_a(CH)]

[a]Experimental intensities (I) are given in qualitative terms: s = strong; m = medium; w = weak; vw = very weak; ov = overlapped. The spectral range in 1900−700 cm^{-1} region plus the ν(N−H) band is reported. [b]Computed harmonic wavenumbers were multiplied by 0.979 [excepted the ν(N−H) mode that was multiplied by 0.960] are expressed in cm^{-1}. [c]PED values lower than 10% are not included. Definition of internal coordinates is given in Table S5.

Table S11. Relative zero-point corrected energies (ΔE_0) computed at the CBS-APNO, B3LYP/6-311+G(2d,p) and M06-2X/6-311++G(d,p) levels of theory for selected reaction pathways of triplet nitrene **s-^{3}2** and **a-^{3}2** conformers.[a]

Structures	CBS-APNO ΔE_0	B3LYP/6-311+G(2d,p) ΔE_0	M06-2X/6-311++G(d,p) ΔE_0
**s-^{3}2**	0.0	0.0	0.0
**TS1**	4.4	5.6	5.3
**a-^{3}2**	0.4	0.5	0.7
**TS2**	15.6	15.7	21.7
**^{3}4**	−2.2	−1.7	0.2

[a]Calculated energies (kcal mol^{-1}) are relative to the **s-^{3}2** species. See also Figure 3.

3. Computational data

Optimized geometries (Cartesian coordinates, Å), electronic energies (E, E_h) and zero-point vibrational energy (ZPVE, E_h) computed at the B3LYP/6-311+G(2d,p), M06-2X/6-311++G(d,p), and CBS-APNO levels of theory.

aa-1: B3LYP/6-311+G(2d,p) (E = -608.574784; ZPVE = 0.103822)

C	1.651347	0.546884	-0.000001
C	0.628744	-0.410387	0.000000
C	-0.696885	0.078913	0.000001
C	-0.959343	1.448482	0.000002
C	0.094407	2.350811	0.000001
C	1.411157	1.908848	0.000000
H	-1.980213	1.809112	0.000003
H	-0.113734	3.413633	0.000002
H	2.247895	2.594149	-0.000001
C	0.889448	-1.872614	0.000000
O	1.982561	-2.388362	-0.000003
H	-0.024734	-2.490211	0.000001
N	-1.727400	-0.890140	0.000002
N	-2.899668	-0.507817	0.000003
N	-4.007534	-0.295623	-0.000002
F	2.926586	0.147739	0.000002

as-1: B3LYP/6-311+G(2d,p) (E = -608.574472; ZPVE = 0.103831)

C	-1.851708	-0.257747	-0.000048
C	-0.683672	0.512293	-0.000091
C	0.541067	-0.196118	0.000046
C	0.545367	-1.594030	0.000215
C	-0.647978	-2.300003	0.000249
C	-1.869703	-1.637301	0.000117
H	1.486094	-2.130162	0.000320
H	-0.626104	-3.382794	0.000381
H	-2.814280	-2.163598	0.000141
C	-0.800206	1.991840	-0.000273
O	0.124672	2.770426	-0.000331
H	-1.840665	2.358559	-0.000358
N	1.725582	0.556449	0.000006
N	2.806790	-0.036983	0.000124
N	3.863839	-0.432014	0.000220
F	-3.041656	0.380979	-0.000175

ss-1: B3LYP/6-311+G(2d,p) (*E* = -608.573920; ZPVE = 0.104107)

C	-1.670368	0.605631	0.146849
C	-0.285995	0.470968	-0.038755
C	0.208934	-0.851712	-0.174398
C	-0.682300	-1.927546	-0.205657
C	-2.043407	-1.726313	-0.048025
C	-2.557205	-0.447742	0.148092
H	-0.277323	-2.922391	-0.332779
H	-2.714369	-2.576236	-0.062298
H	-3.613295	-0.262052	0.288075
C	0.515264	1.698043	-0.221257
O	1.664840	1.723360	-0.607073
H	-0.026309	2.635943	-0.020477
N	1.557504	-1.220314	-0.324936
N	2.467570	-0.608921	0.248368
N	3.399983	-0.203839	0.727799
F	-2.174707	1.849045	0.309373

a-^{3}2: B3LYP/6-311+G(2d,p) (*E* =-499.010796; ZPVE = 0.091641)

C	0.751054	1.183096	0.000102
C	-0.404137	0.308545	0.000016
C	-0.165528	-1.057800	-0.000089
C	1.116080	-1.603117	-0.000110
C	2.225271	-0.753716	-0.000019
C	2.065495	0.614126	0.000089
N	0.627022	2.493642	0.000215
C	-1.748038	0.927985	0.000043
O	-2.796025	0.327450	-0.000022
H	-1.721646	2.034713	0.000131
F	-1.188760	-1.916606	-0.000176
H	1.223563	-2.679743	-0.000194
H	3.220895	-1.179984	-0.000032
H	2.913895	1.284659	0.000162

s-^{3}2: B3LYP/6-311+G(2d,p) (*E* = -499.005378; ZPVE = 0.091871)

C	-1.181019	-0.334019	0.000000
C	0.000000	0.494434	0.000000
C	1.227654	-0.149338	0.000000
C	1.374301	-1.529717	0.000000
C	0.228479	-2.325339	0.000000
C	-1.027383	-1.753757	0.000000
N	-2.406811	0.158286	0.000000
C	-0.095294	1.965490	0.000000
O	-1.143007	2.571000	0.000000
H	0.869812	2.500137	0.000000
F	2.353981	0.593225	0.000000
H	2.368338	-1.956537	0.000000
H	0.329374	-3.403628	0.000000
H	-1.922039	-2.361518	0.000000

3: B3LYP/6-311+G(2d,p) (*E* = -499.049889; ZPVE = 0.096450)

C	-0.987275	-0.473142	0.000000
C	0.000000	0.571935	0.000000
C	1.370561	0.221390	0.000000
C	1.743451	-1.081766	0.000000
C	0.743593	-2.110637	0.000000
C	-0.592625	-1.842351	0.000000
N	-2.214802	0.021532	0.000000
C	-0.743787	1.719870	0.000000
O	-2.039272	1.406575	0.000000
H	-0.499153	2.768928	0.000000
F	2.286162	1.211263	0.000000
H	2.794331	-1.338890	0.000000
H	1.080746	-3.140003	0.000000
H	-1.337095	-2.626523	0.000000

^{3}**3**: B3LYP/6-311+G(2d,p) (*E* = -498.971455; ZPVE = 0.091973)

C	-0.961164	-0.479864	0.000000
C	0.000000	0.589459	0.000000
C	1.340427	0.246655	0.000000
C	1.756778	-1.120075	0.000000
C	0.826974	-2.120133	0.000000
C	-0.576717	-1.817267	0.000000
N	-2.222177	-0.007548	0.000000
C	-0.766919	1.742441	0.000000
O	-2.109886	1.410788	0.000000
H	-0.532420	2.792417	0.000000
F	2.289684	1.201553	0.000000
H	2.818344	-1.330952	0.000000
H	1.145410	-3.153878	0.000000
H	-1.320444	-2.602329	0.000000

^{3}**4**: B3LYP/6-311+G(2d,p) (*E* = -499.014742; ZPVE = 0.092227)

C	-0.796048	1.135231	0.000004
C	0.397551	0.312081	-0.000002
C	0.248614	-1.067464	0.000001
C	-0.997323	-1.688483	-0.000002
C	-2.151635	-0.897190	-0.000007
C	-2.065300	0.477106	-0.000003
N	-0.786066	2.461324	0.000009
C	1.705228	0.977496	-0.000003
O	2.800250	0.525937	-0.000009
H	0.184158	2.795487	0.000004
F	1.331558	-1.853205	0.000011
H	-1.045530	-2.769647	0.000002
H	-3.121255	-1.379578	-0.000009
H	-2.947460	1.103175	-0.000007

4: B3LYP/6-311+G(2d,p) (*E* =-499.056830; ZPVE = 0.094103)

C	1.357672	-0.172094	0.000000
C	0.000000	0.471947	0.000000
C	-1.193018	-0.340991	0.000000
C	-1.152741	-1.679225	0.000000
C	0.141981	-2.319310	0.000000
C	1.301070	-1.629118	0.000000
N	2.498436	0.419247	0.000000
C	-0.107609	1.799541	0.000000
O	-0.139452	2.949432	0.000000
H	2.432841	1.437262	0.000000
F	-2.361178	0.337166	0.000000
H	-2.068240	-2.253787	0.000000
H	0.168433	-3.402400	0.000000
H	2.260004	-2.130254	0.000000

TS1: B3LYP/6-311+G(2d,p) (*E* = -499.001908; ZPVE = 0.090970)

C	-1.740597	1.176798	-0.079852
C	-0.322317	1.278860	0.083919
C	0.459250	0.074904	0.145552
C	-0.203908	-1.125869	0.067541
C	-1.583208	-1.230336	-0.091866
C	-2.342387	-0.059917	-0.166707
H	-2.315885	2.091294	-0.131881
H	-2.036074	-2.210847	-0.154884
H	-3.415656	-0.129342	-0.292197
C	1.957991	0.136573	0.305672
N	0.252979	2.463280	0.188826
O	2.708954	0.224983	-0.626846
H	2.336078	0.095393	1.345677
F	0.515567	-2.266156	0.148969

TS2: B3LYP/6-311+G(2d,p) (*E* = -498.982201; ZPVE = 0.087345)

C	0.366404	1.277047	0.000104
C	-0.433651	0.073933	0.000011
C	0.175569	-1.163996	-0.000096
C	1.563093	-1.270559	-0.000113
C	2.346174	-0.105694	-0.000019
C	1.783026	1.155891	0.000087
N	-0.300887	2.428639	0.000194
C	-1.836556	0.499119	0.000052
O	-2.870851	-0.083761	0.000011
H	-1.516234	1.835592	0.000167
F	-0.558700	-2.283158	-0.000188
H	2.016960	-2.253043	-0.000198
H	3.424548	-0.208830	-0.000034
H	2.391703	2.049875	0.000157

MECP: B3LYP/6-311+G(2d,p) (*E* = -498.995042; ZPVE = 0.090845)

C	0.332687	-1.037230	-0.001342
C	0.544165	0.391228	-0.000578
C	-0.967710	0.894658	-0.000641
C	-2.068734	0.072972	0.000444
C	-1.864491	-1.323607	0.001621
C	-0.614345	-1.889960	0.000251
F	-1.134537	2.231943	-0.001611
H	-0.475140	-2.961402	0.000974
N	1.758691	-1.509857	-0.002305
C	1.572319	1.093098	0.002014
O	2.620318	0.439882	0.001075
H	1.603530	2.189524	0.005993
H	-2.732298	-1.973002	0.003841
H	-3.061979	0.500385	0.000613

5: B3LYP/6-311+G(2d,p) (*E* = -499.073008; ZPVE = 0.095568)

C	0.230931	-1.123125	-0.026966
C	0.303940	0.273883	-0.032129
C	-0.839065	1.027515	-0.019313
C	-2.069292	0.358712	0.020804
C	-2.105036	-1.030643	0.044035
C	-0.942649	-1.829296	0.018812
N	1.652929	-1.307976	-0.147419
C	1.813308	0.131778	-0.016592
O	2.785866	0.804470	0.093791
H	2.192176	-1.951675	0.420960
F	-0.816666	2.367487	-0.024424
H	-2.981900	0.939510	0.038972
H	-3.071612	-1.519028	0.077748
H	-0.998913	-2.909072	0.031836

a-^{3}2: ROM06-2X/6-311++G(d,p) (*E* = -498.791860; ZPVE = 0.092966)

C	0.757844	1.161788	0.000107
C	-0.389962	0.315245	0.000018
C	-0.170060	-1.056488	-0.000089
C	1.104028	-1.608719	-0.000109
C	2.213845	-0.768379	-0.000020
C	2.056906	0.606088	0.000088
N	0.635580	2.503307	0.000215
C	-1.742939	0.935366	0.000043
O	-2.775629	0.322944	-0.000023
H	-1.731431	2.040320	0.000125
F	-1.200955	-1.894124	-0.000178
H	1.202312	-2.686698	-0.000195
H	3.208061	-1.197779	-0.000035
H	2.907642	1.275166	0.000158

s-^{3}2: ROM06-2X/6-311++G(d,p) (*E* = -498.793023; ZPVE = 0.093017)

C	-1.170432	-0.329695	0.000000
C	0.000000	0.483777	0.000000
C	1.226705	-0.162359	0.000000
C	1.364002	-1.541681	0.000000
C	0.213228	-2.324392	0.000000
C	-1.041041	-1.737157	0.000000
N	-2.415589	0.185535	0.000000
C	-0.075973	1.965448	0.000000
O	-1.112514	2.574765	0.000000
H	0.895351	2.486407	0.000000
F	2.344412	0.578460	0.000000
H	2.356089	-1.973851	0.000000
H	0.302348	-3.403701	0.000000
H	-1.943183	-2.335511	0.000000

^{3}3: ROM06-2X/6-311++G(d,p) (*E* = -498.747326; ZPVE = 0.092195)

C	-0.940462	-0.496154	0.000000
C	0.000000	0.580588	0.000000
C	1.338541	0.262989	0.000000
C	1.768209	-1.090830	0.000000
C	0.848349	-2.104174	0.000000
C	-0.553767	-1.817137	0.000000
N	-2.223086	-0.023944	0.000000
C	-0.788995	1.731468	0.000000
O	-2.124102	1.369454	0.000000
H	-0.573670	2.785045	0.000000
F	2.264054	1.228301	0.000000
H	2.832872	-1.287576	0.000000
H	1.180531	-3.133690	0.000000
H	-1.293051	-2.607011	0.000000

3: ROM06-2X/6-311++G(d,p) (*E* = -498.841798; ZPVE = 0.097941)

C	-0.986850	-0.466718	0.000000
C	0.000000	0.573635	0.000000
C	1.376052	0.221569	0.000000
C	1.744328	-1.077808	0.000000
C	0.738202	-2.109840	0.000000
C	-0.593296	-1.843253	0.000000
N	-2.205605	0.032767	0.000000
C	-0.748344	1.716707	0.000000
O	-2.033585	1.391344	0.000000
H	-0.513034	2.768183	0.000000
F	2.278953	1.209237	0.000000
H	2.795384	-1.334569	0.000000
H	1.076294	-3.138960	0.000000
H	-1.341863	-2.623657	0.000000

[3]**4**: ROM06-2X/6-311++G(d,p) (*E* = -498.792682; ZPVE = 0.093024)

C	0.808632	1.124339	0.000000
C	-0.386425	0.318922	-0.000001
C	-0.263475	-1.061374	0.000000
C	0.971745	-1.699103	0.000001
C	2.134880	-0.925023	0.000002
C	2.066683	0.451671	0.000002
N	0.823513	2.450658	0.000000
C	-1.698183	0.992183	-0.000002
O	-2.784079	0.540890	-0.000003
H	-0.138580	2.803568	-0.000001
F	-1.356857	-1.818155	-0.000001
H	1.001496	-2.781270	0.000002
H	3.098295	-1.419713	0.000003
H	2.955406	1.069397	0.000003

4: ROM06-2X/6-311++G(d,p) (*E* = -498.847994; ZPVE = 0.095546)

C	1.356898	-0.159495	0.000000
C	0.000000	0.467452	0.000000
C	-1.194790	-0.348287	0.000000
C	-1.148917	-1.682772	0.000000
C	0.156303	-2.316810	0.000000
C	1.309274	-1.623254	0.000000
N	2.488806	0.436067	0.000000
C	-0.114770	1.794805	0.000000
O	-0.157167	2.937388	0.000000
H	2.407551	1.452763	0.000000
F	-2.350502	0.331257	0.000000
H	-2.062597	-2.259999	0.000000
H	0.188109	-3.399832	0.000000
H	2.273170	-2.115649	0.000000

TS1: ROM06-2X/6-311++G(d,p) (*E* = -498.784106; ZPVE = 0.092468)

C	-1.716851	1.191405	-0.079502
C	-0.315928	1.265054	0.083634
C	0.450284	0.075810	0.152667
C	-0.216333	-1.128049	0.070035
C	-1.591273	-1.217734	-0.090901
C	-2.336129	-0.043296	-0.166733
H	-2.281191	2.113536	-0.134831
H	-2.051918	-2.195078	-0.155074
H	-3.409814	-0.100357	-0.294054
C	1.950156	0.130479	0.306988
N	0.294889	2.465978	0.183088
O	2.688334	0.186072	-0.632335
H	2.333637	0.118997	1.343181
F	0.499426	-2.258837	0.151192

TS2: ROM06-2X/6-311++G(d,p) (E = -498.753730; ZPVE = 0.088242)

C	-0.343320	-1.269279	0.000124
C	0.424851	-0.065215	0.000041
C	-0.201217	1.166024	-0.000117
C	-1.586396	1.245383	-0.000113
C	-2.343810	0.067738	0.000182
C	-1.753491	-1.184661	0.000320
N	0.352949	-2.420460	-0.000205
C	1.842872	-0.470733	-0.000169
O	2.861515	0.122027	0.000361
H	1.553759	-1.799943	-0.000406
F	0.518744	2.284063	-0.000339
H	-2.057000	2.220232	-0.000349
H	-3.424215	0.147781	0.000260
H	-2.340934	-2.093190	0.000480

MECP: ROM06-2X/6-311++G(d,p) (E = -498.775845; ZPVE = 0.091235)

C	-0.2357677	-0.0178148	-0.0088953
C	0.9560286	-0.8126930	0.0000418
C	0.8662993	-2.2034564	0.0389575
C	-0.3425079	-2.8500605	0.0683041
C	-1.5182419	-2.0672654	0.0581368
C	-1.5002946	-0.6960050	0.0214617
F	2.0056615	-2.9056791	0.0469669
H	-2.4103351	-0.1131461	0.0143006
N	-0.1432602	1.2882241	-0.0449016
C	2.1127656	0.0290406	-0.0371948
O	1.9294826	1.2413481	-0.0677009
H	3.1262472	-0.3885235	-0.0394601
H	-2.4753749	-2.5750404	0.0802116
H	-0.3768635	-3.9301285	0.0979236

a-^{3}2: CBS-APNO (E = -498.836659; ZPVE = 0.088943)

C	0.761459	1.182702	0.000110
C	-0.398194	0.313493	0.000018
C	-0.174475	-1.057547	-0.000089
C	1.112447	-1.612673	-0.000109
C	2.234988	-0.766003	-0.000019
C	2.080805	0.608643	0.000089
N	0.637723	2.503731	0.000214
C	-1.763356	0.936130	0.000042
O	-2.802328	0.324163	-0.000025
H	-1.741116	2.043528	0.000130
F	-1.200211	-1.908596	-0.000177
H	1.211726	-2.693208	-0.000194
H	3.230403	-1.200700	-0.000035
H	2.933391	1.279857	0.000159

s-^{3}2: CBS-APNO (E = -498.837268; ZPVE = 0.088978)

C	-1.188805	-0.336308	0.000000
C	0.000000	0.491150	0.000000
C	1.229686	-0.148373	0.000000
C	1.377953	-1.540434	0.000000
C	0.224240	-2.342827	0.000000
C	-1.035652	-1.767938	0.000000
N	-2.418566	0.161955	0.000000
C	-0.086046	1.983977	0.000000
O	-1.127323	2.594804	0.000000
H	0.889814	2.502084	0.000000
F	2.351435	0.592712	0.000000
H	2.376282	-1.964944	0.000000
H	0.326563	-3.424165	0.000000
H	-1.935281	-2.374989	0.000000

^{3}4: CBS-APNO (E = -498.841849; ZPVE = 0.090105)

C	0.805245	1.137593	0.000000
C	-0.392940	0.313805	0.000000
C	-0.259036	-1.067745	0.000000
C	0.992762	-1.699835	0.000001
C	2.160420	-0.910853	0.000002
C	2.081249	0.469447	0.000002
N	0.804845	2.466595	0.000000
C	-1.721220	0.993439	-0.000002
O	-2.810663	0.525952	-0.000003
H	-0.168423	2.791841	-0.000001
F	-1.345260	-1.843500	-0.000001
H	1.032175	-2.784449	0.000002
H	3.129497	-1.401811	0.000003
H	2.966588	1.097046	0.000003

TS1: CBS-APNO (E =-498.830167; ZPVE =0.088968)

C	-1.754063	1.174898	-0.080205
C	-0.331079	1.281964	0.085513
C	0.457873	0.079023	0.147024
C	-0.195225	-1.132927	0.069042
C	-1.582414	-1.245455	-0.092586
C	-2.352730	-0.072004	-0.165836
H	-2.335585	2.089742	-0.133096
H	-2.029793	-2.231842	-0.155229
H	-3.428716	-0.147849	-0.292202
C	1.961864	0.146133	0.309192
N	0.249451	2.475120	0.184223
O	2.711910	0.225328	-0.630602
H	2.333674	0.118402	1.352015
F	0.532626	-2.260744	0.150100

TS2: CBS-APNO (*E* =-498.808370; ZPVE =0.084875)

C	-0.355412	-1.278406	0.000103
C	0.428822	-0.065584	0.000008
C	-0.182853	1.173670	-0.000098
C	-1.580036	1.267193	-0.000114
C	-2.359280	0.087812	-0.000022
C	-1.783817	-1.173464	0.000086
N	0.322885	-2.430198	0.000203
C	1.852348	-0.495656	0.000052
O	2.882545	0.096434	0.000010
H	1.516014	-1.830930	0.000160
F	0.545860	2.291372	-0.000186
H	-2.043195	2.248790	-0.000198
H	-3.441572	0.184022	-0.000036
H	-2.383174	-2.077718	0.000157

1': B3LYP/6-311+G(2d,p) (*E* = -509.3117747; ZPVE = 0.112201)

C	-1.948785	0.599365	0.000001
C	-0.931742	-0.359787	0.000000
C	0.407760	0.068050	0.000000
C	0.704135	1.433581	0.000000
C	-0.323949	2.364179	0.000000
C	-1.656572	1.952224	0.000000
H	-2.971650	0.243652	0.000000
H	1.734787	1.767863	0.000000
H	-0.081886	3.420288	0.000000
H	-2.453654	2.684784	0.000000
C	-1.291621	-1.797922	0.000002
O	-2.434431	-2.200957	0.000002
H	-0.445435	-2.505427	0.000004
N	1.407868	-0.934468	-0.000001
N	2.591627	-0.591012	-0.000002
N	3.705924	-0.411898	-0.000002

a-^{3}2': B3LYP 6-311+G(2d,p) (*E* = -399.747794; ZPVE = 0.099962)

C	-0.372834	1.072321	-0.000103
C	0.546718	-0.041685	0.000001
C	0.051445	-1.336962	0.000109
C	-1.318690	-1.580985	0.000128
C	-2.226662	-0.510161	0.000043
C	-1.780419	0.790405	-0.000065
N	0.038413	2.321895	-0.000189
C	2.003775	0.212695	-0.000014
O	2.837203	-0.665962	0.000063
H	2.297567	1.278364	-0.000105
H	0.762777	-2.153842	0.000179
H	-1.686327	-2.599409	0.000215
H	-3.291694	-0.709609	0.000065
H	-2.468825	1.625154	-0.000128

^{3}4': B3LYP 6-311+G(2d,p) (*E* = -399.748653; ZPVE = -399.748653)

C	-0.332137	1.051784	0.000001
C	0.556054	-0.086156	0.000001
C	0.048219	-1.380001	0.000000
C	-1.327100	-1.597192	-0.000002
C	-2.213994	-0.507417	-0.000002
C	-1.738202	0.782586	-0.000002
N	0.060716	2.320567	0.000001
C	2.010658	0.146095	0.000002
O	2.891270	-0.647891	0.000002
H	1.085901	2.358667	0.000001
H	0.738361	-2.215134	0.000000
H	-1.713199	-2.609186	-0.000002
H	-3.282215	-0.688632	-0.000004
H	-2.405013	1.635243	-0.000002

4': B3LYP/6-311+G(2d,p) (*E* = -399.787731; ZPVE = 0.102166)

C	-0.073453	1.047698	-0.000002
C	0.496493	-0.337522	-0.000002
C	-0.355801	-1.514958	0.000001
C	-1.694983	-1.369687	0.000002
C	-2.282268	-0.051283	0.000001
C	-1.531456	1.069711	-0.000003
N	0.568647	2.163437	0.000004
C	1.819036	-0.487478	-0.000001
O	2.968077	-0.585327	0.000000
H	1.582702	2.048093	0.000006
H	0.105518	-2.494335	0.000000
H	-2.336075	-2.241528	0.000002
H	-3.363058	0.032243	0.000003
H	-1.979636	2.055204	-0.000006

TS2': B3LYP/6-311+G(2d,p) (*E* = -399.715683; ZPVE = 0.095489)

C	-0.167452	1.016821	-0.000082
C	0.463547	-0.278170	0.000012
C	-0.262446	-1.454476	0.000119
C	-1.654725	-1.369130	0.000135
C	-2.299475	-0.117449	0.000045
C	-1.590448	1.066292	-0.000061
N	0.639515	2.076264	-0.000178
C	1.912939	-0.034665	-0.000026
O	2.853279	-0.762159	0.000026
H	1.767542	1.337461	-0.000150
H	0.238855	-2.414829	0.000189
H	-2.249847	-2.273922	0.000218
H	-3.382836	-0.087822	0.000061
H	-2.088197	2.027200	-0.000130

4. References

(S1) Nunes, C. M.; Reva, I.; Kozuch, S.; McMahon, R. J.; Fausto, R. Photochemistry of 2-Formylphenylnitrene: A Doorway to Heavy-Atom Tunneling of a Benzazirine to a Cyclic Ketenimine. *J. Am. Chem. Soc.* **2017**, *139*, 17649–17659.

(S2) Nunes, C. M.; Knezz, S. N.; Reva, I.; Fausto, R.; McMahon, R. J. Evidence of a Nitrene Tunneling Reaction: Spontaneous Rearrangement of 2-Formyl Phenylnitrene to an Imino Ketene in Low-Temperature Matrixes. *J. Am. Chem. Soc.* **2016**, *138*, 15287–15290.

(S3) Nunes, C. M.; Viegas, L. P.; Wood, S. A.; Roque, J. P. L.; McMahon, R. J.; Fausto, R. Heavy-Atom Tunneling Through Crossing Potential Energy Surfaces: Cyclization of a Triplet 2-Formylarylnitrene to a Singlet 2,1-Benzisoxazole. *Angew. Chem. Int. Ed. Engl.* **2020**, *59*, 17622−17627.
